# HPLC/GC–MS and Electronic Sensing Reveal Tissue-Wide Differences in Bioactive and Flavor Compound Distribution in Coffee Fruits Across Multiple Varieties

**DOI:** 10.3390/foods15020269

**Published:** 2026-01-12

**Authors:** Lu-Xia Ran, Xiao-Hua Dai, Er-Fang Ren, Jin-Hong Li, Lin Yan, Usman Rasheed, Gan-Lin Chen

**Affiliations:** 1 Guangxi Subtropical Crops Research Institute, Guangxi Academy of Agricultural Sciences, Nanning 530001, China; 1211016008@gnnu.edu.cn (L.-X.R.); xiaohuadai2023@163.com (X.-H.D.); aabbc159@163.com (E.-F.R.); rasheus@outlook.com (U.R.); 2 Key Laboratory of Quality and Safety Control for Subtropical Fruit and Vegetable, Ministry of Agriculture and Rural Affairs, Nanning 530001, China; 3 Guangxi Key Laboratory of Quality and Safety Control for Subtropical Fruits, Nanning 530001, China; 4 Yunnan Dehong Institute of Tropical Agricultural Science, Ruili 678600, China; lijinhong1969@hotmail.com; 5 Spice and Beverage Research Institute, Chinese Academy of Tropical Agricultural Sciences, Wanning 571533, China; yanlin2575@163.com

**Keywords:** green coffee beans, coffee byproducts, bioactive compounds, volatile compounds

## Abstract

The quality of different coffee varieties varies, and the corresponding bioactive value of coffee processing byproducts is often overlooked. For that, we employed HPLC, GC-MS, and electronic sensory analyses to evaluate the key bioactive components, antioxidant potential, and flavor traits of green coffee bean and coffee processing byproducts of seven coffee varieties. The results showed that green coffee beans (Oe+Ie) and exocarp (Ep) possessed strong antioxidant activity and high total phenolic content (TPC), caffeine and trigonelline content. Among the varieties, DR390 contained higher levels of total phenols, caffeine, and trigonelline, whereas DR402 was rich in caffeine and chlorogenic acid. In addition, RY3 exhibited higher TPC, total flavonoid content (TFC), caffeine, and chlorogenic acid. The parchment (Pc) layer was rich in soluble sugars (1.83–5.43%), while the silverskin (Sk) contained relatively high levels of chlorogenic acid (3.58–4.69 mg/g). Flavor analysis identified eleven classes of volatile compounds in green coffee bean (Oe+Ie) and byproducts (Ep, Pc, Sk), with esters, ketones, alcohols, and aldehydes being the most prevalent. Seven key aroma compounds, including methyl salicylate, phenethyl alcohol, nonanal, and benzaldehyde, were identified across the various structural tissues of coffee fruit. Distinct flavor profiles were observed among the coffee fruit parts: green coffee bean (Oe+Ie) was nutty; the Ep showed fruity and cocoa-like aromas; the Pc and Sk exhibited papery and nutty aromas, respectively. Varieties DR397, DR402, and RY3 exhibited pronounced aroma profiles. Comprehensive analysis showed that DR402 and RY3 had higher overall scores for bioactive and flavor components than other varieties in their groups. In summary, green coffee bean (Oe+Ie) exhibited strong antioxidant activity and high levels of bioactive compounds. Coffee byproducts, such as the Ep, hold potential for extracting natural antioxidants and bioactive compounds to develop specialty products or for other high-value utilization.

## 1. Introduction

Coffee (*Coffea* spp.), belonging to the genus *Coffea* within the family Rubiaceae, is an evergreen shrub native to tropical and subtropical areas of northern and central Africa. In China, coffee farming is mainly concentrated in Yunnan, Hainan, and Guangxi provinces, with *Coffea arabica* and *Coffea canephora* as the predominant varieties [1].

The chemical composition of green coffee beans is essential to coffee quality; volatile compounds contribute to the distinctive aroma of coffee, while bioactive constituents form the essential foundation of its taste and mouthfeel. Studies have shown that coffee beans contain more than 1000 compounds, including carbohydrates, lipids, and nitrogen-containing organic compounds. These constituents undergo complex biochemical transformations during roasting to produce distinctive fruity acids and other flavor compounds [2]. Green coffee beans contain high contents of chlorogenic acid, caffeine, and trigonelline. Chlorogenic acid accounts for approximately 6% to 12% [3] of the coffee’s mass and exists in coffee in the form of several isomers. Caffeoylquinic acids (CQAs) are the most abundant, including neochlorogenic acid and cryptochlorogenic acid, followed by dicaffeoylquinic acids (diCQAs) such as 3,5-dicaffeoylquinic acid, 3,4-dicaffeoylquinic acid, and 4,5-dicaffeoylquinic acid [4]. Coffee chlorogenic acids have antioxidant and anti-inflammatory effects [5], regulate sugar and lipid metabolism, protect the nervous system [6], and contribute to coffee bitterness [7]. Caffeine has refreshing effects and stimulates the central nervous system [8]. It can also inhibit obesity and lower blood glucose [9,10], and it is an important flavor component in coffee. In addition, trigonelline has neuroprotective and antidiabetic effects [11]. After roasting, coffee beans undergo chemical reactions, including Maillard reactions and caramelization, involving chlorogenic acids, trigonelline, polysaccharides, fats, and proteins. These reactions generate pyrazines, furans, pyrroles, pyrans, pyridines, and many aldehydes, ketones, phenols, esters, and alcohols. These compounds contribute to the unique flavor of coffee [12,13,14]. Gallardo-Ignacio et al. [15] compared the chemical properties of Arabica green, medium, and dark roasted beans and found that chlorogenic acid (55 mg/g) and caffeine (1.8 mg/g) were relatively high in green beans and that caffeine and melanoidin increased with roasting degree. Mehari et al. [16] compared the polyphenol content of green coffee beans from different geographical origins and found significant variations in polyphenol contents with Harar coffee exhibiting the lowest total polyphenol content. Previous research on coffee flavor characteristics primarily examined the changes occurring during coffee fermentation [17] and roasting [18]. Different coffee beans vary in their bioactive composition and flavor, and these differences impact coffee quality and economic value. Therefore, analyzing the bioactive and flavor profiles of different green coffee beans can help assess coffee quality and offer useful references for breeding coffee varieties.

In the coffee processing industry, coffee beans are the main commodity used for deep processing, while approximately 90% of coffee byproducts (exocarp, parchment, silverskin) are discarded and considered agricultural waste [19], thereby leading to substantial environmental issues and resource waste [20]. Studies have shown that these byproducts make up approximately 40–60% of the coffee fruit mass [21], and contain a variety of bioactive compounds such as polyphenols, alkaloids, chlorogenic acids, caffeine, anthocyanins, trace elements [22], carbohydrates (58–85%), and cellulose (24.5%) [23]. Coffee exocarp is a major byproduct of coffee processing and is rich in polyphenols and polysaccharides [24,25]. The European Food Safety Authority (EFSA) has approved the use of coffee husk as a food ingredient [23]. It has been used in products like cascara tea [26], biscuits [27], and active coating [24], to enhance the content of bioactive and antioxidant compounds. Coffee parchment accounts for approximately 2.10% of the coffee bean and consists of about 35% xylan, 32% lignin, and 12% cellulose, making it a potential source of insoluble dietary fiber [28]. Coffee parchment also shows antioxidant, hypoglycemic, and hypolipidemic activities [29]. Coffee silverskin contains high levels of total phenolics, caffeine [30], minerals, chlorogenic acid and its derivatives [31]. As a supplement, it can increase the phenolic content and antioxidant capacity of foods [32]. In addition, coffee silverskin has low moisture content and is more stable during storage [31]. Machado et al. found that ash, protein, fat, and total dietary fiber contents were significantly higher in defective beans and parchment than in other coffee byproducts (*p* < 0.05). All coffee byproducts can serve as potential sources of 5-caffeoylquinic acid [33]. Therefore, valorizing coffee processing byproducts can reduce environmental risks and enable their conversion into nutritious foods or functional ingredients, supporting the sustainable development of the coffee industry.

Given the aforementioned literature, research on the chemical composition of different coffee bean varieties and their byproducts remains limited, and the flavor profiles of these byproducts have not yet been documented. Therefore, this study employed HPLC, GC-MS, and electronic sensory analysis to characterize the bioactive compounds, antioxidant capacities, and volatile flavor substances of seven different coffee varieties and their structural tissues. The aim was to elucidate the nutritional and flavor differences among various coffee beans and their byproducts, offering a reference for selecting high-quality coffee varieties and exploring the potential uses of coffee byproducts.

## 2. Materials and Methods

### 2.1. Materials

**Figure 1:**
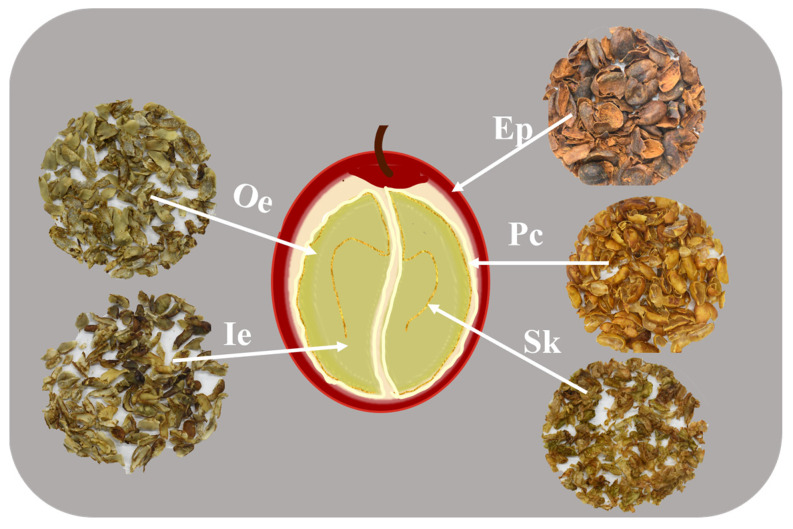
Anatomical tissues evaluated in coffee fruit.

In this study, seven coffee materials were selected, and 10 kg of each was sampled as a mixed sample from the fruits of five coffee trees. Out of which 5 *Coffea arabica* (Arabica coffee) varieties: De’re 390, De’re 394, De’re 397, De’re 401, and De’re 402 (abbreviated as DR390, DR394, DR397, DR401, and DR402) were the newly developed varieties by Dehong Tropical Agricultural Science Institute in Yunnan Province. In addition, *Coffea canephora* (Robusta coffee) varieties Reyan No.3 (RY3) and Reyan No.5 (RY5) were supplied by Spices and Beverage Research Institute of the Chinese Academy of Tropical Agricultural Sciences. Each of the coffee berry samples was dissected into five anatomical parts: exocarp (Ep), parchment (Pc), silverskin (Sk), outer endosperm (Oe), and inner endosperm (Ie) (Figure 1). Among them, Ep, Pc and Sk represented byproducts, while Oe and Ie were considered green coffee beans. All the samples were dried in an electric thermostatic drying oven (Shanghai Jinghong Laboratory Equipment Co., Ltd., DHG-9240A, Shanghai, China) at 40 °C until constant weight and preserved at −20 °C for further analysis (unless otherwise specified, this dried sample will be used in all subsequent experiments).

### 2.2. Determination of Fresh Weight and Moisture Content

The fresh weight of each part of the coffee fruit (Ep, Pc, Sk, Oe, Ie) was measured, and the fresh weight percentage (FW%) was calculated. Each part was then dried using an electric thermostatic drying oven (Shanghai Jinghong Laboratory Equipment Co., Ltd., DHG-9240A) at 40 °C to a constant weight, and the dry weight (g) was recorded to determine the moisture content (%).

### 2.3. Measurement of Antioxidant Activity

In vitro antioxidant activity was evaluated using 2,2-diphenyl-1-picrylhydrazyl (DPPH•) and 2,2′-azinobis(3-ethylbenzothiazoline-6-sulfonic acid) radical cation (ABTS+•) scavenging assays and the ferric reducing antioxidant power (FRAP) assay, following a previously developed method [34] with minor modifications. Briefly, 0.5 g of dried sample was mixed with 15 mL of 70% ethanol solution, ultrasonically extracted for 60 min, centrifuged, and the supernatant was collected for subsequent analyses.

To determine the DPPH• scavenging rate, three centrifuge tubes (A_0_, A_1_, A_2_) were prepared: A0 with 1 mL ethanol and 1 mL 0.2 mmol/L DPPH; A_1_ with 1 mL sample and 1 mL DPPH•; A_2_ with 1 mL sample and 1 mL ethanol. After thorough mixing, the mixtures were incubated in the dark for 30 min, and the absorbance was measured at 517 nm. The DPPH• scavenging rate (%) was calculated according to Equation (1).

(1) $$DPPH•scavenging\;rate\;(\%)=1-(\frac{A_{1}-A_{2}}{A_{0}})\times 100$$

For ABTS+• scavenging rate, the ABTS working solution was prepared by mixing 0.2 mL of 7.4 mmol/L ABTS stock solution with 0.2 mL of 2.6 mmol/L K_2_S_2_O_8_, followed by incubation in the dark at room temperature for 12 h. The resulting mixture was diluted with absolute ethanol until its absorbance at 734 nm reached 0.70 ± 0.02. A mixture of 1 mL of the sample solution and 1 mL of the ABTS working solution was incubated in the dark for 6 min. Afterwards, absorbance was measured at 734 nm and recorded as “A” sample. Absolute ethanol (1 mL) served as a blank control instead of the sample, with its absorbance recorded as “B”. The scavenging rate (%) was then calculated using the following Equation (2).

(2) $$ABTS+•\;scavenging\;rate\;(\%)=(\frac{B-A}{B})\times 100$$

The ferric-reducing antioxidant potential (FRAP) assay was performed following a procedure described in a previous study [35] with some modifications. The FRAP working solution was freshly prepared by mixing acetate buffer (300 mmol/L, pH 3.6), 2,4,6-Tris(2-pyridyl)-1,3,5-triazine (TPTZ) solution (10 mmol/L, in 40 mmol/L hydrochloric acid), and FeCl_3_ solution (20 mmol/L, in water) in a 10:1:1 volume ratio, and it was kept in a 37 °C water bath before use. A range of FeSO_4_·7H_2_O standard solutions at various concentrations (0–60 µg/mL) was prepared, and 500 µL of each was combined with 1.5 mL of the FRAP working solution. After incubating in the dark water bath (approximately 37 °C) for 15 min, the absorbance was measured at 593 nm, and a standard curve was plotted (y = 0.3649x − 0.3583, R^2^ = 0.9973). Next, 500 µL of the sample solution was combined with 1.5 mL of the FRAP working solution, incubated in the dark under the same conditions, and measured at 593 nm. The Fe^2+^ reducing ability of the sample was expressed as the equivalent FeSO_4_ concentration (µg/mL), determined from the standard curve.

### 2.4. Determination of Soluble Sugar, Total Phenolic, and Flavonoid Contents

The method using 3,5-dinitrosalicylic acid (DNS) reported in reference [36], for determining soluble sugar content, was slightly modified. Briefly, 0.10 g of the dried sample was mixed with a measured amount of distilled water, and then incubated in a water bath at 80 °C for 10 min. The mixture was subsequently diluted to a final volume of 50 mL (V_1_) with distilled water and filtered for analysis. To create the glucose standard curve, 2 mL of glucose standard solutions at various concentrations (0–0.12 mg/mL) were each combined with 4 mL of DNS reagent and heated in a boiling water bath for 5 min. After cooling to room temperature, the reaction mixtures were diluted to 10 mL with distilled water, and the absorbance was measured at 540 nm. A standard curve was plotted with glucose concentration (x, mg/mL) on the x-axis and absorbance (y) on the y-axis, yielding the regression equation y = 0.0103x − 0.0262 (R^2^ = 0.9949). The soluble sugar contents in the samples were determined using the same procedure as that used to generate the standard curve.

Total phenolic content (TPC) was determined using a slightly modified Folin–Ciocalteu method based on a previously reported procedure [37]. TPC extraction was performed by extracting 1.0 g of dried sample with 30 mL of 70% (*v*/*v*) ethanol, followed by ultrasonic extraction for 90 min. The filtrate was used for the analysis of total phenolics. To prepare the standard curve, mix 0.1 mL of gallic acid standard solutions at different concentrations with 0.5 mL of 10% (*v*/*v*) Folin–Ciocalteu reagent and let sit for 5 min. Then, 2 mL of 7.5% (*w*/*v*) Na_2_CO_3_ solution was mixed thoroughly, and the reaction was kept in the dark at 40 °C for 30 min. The absorbance was measured at 765 nm, and a standard curve was plotted against gallic acid concentration. The sample analysis employed the same procedure as the standard solutions, and the phenolic concentration (C, µg/mL) was determined from the standard curve. Results were expressed as milligrams of gallic acid equivalents per gram of sample (mg GAE/g).

The total flavonoid content (TFC) was measured using the sodium nitrite–aluminum nitrate colorimetric method [38], and sample extraction was carried out as described previously for TPC determination. For the standard curve, 0.4 mL of rutin standards at various concentrations was pipetted and mixed with 3.6 mL of 50% ethanol and 0.4 mL of 5% sodium nitrite solution. After standing for 6 min, 0.4 mL of 10% aluminum nitrate [Al (NO_3_)_3_] solution was added, followed by another 6 min. of incubation. Subsequently, 4 mL of 4% sodium hydroxide (NaOH) solution was added, and the mixture was diluted to 10 mL with 50% ethanol. After thorough mixing, the reaction was allowed to proceed for 15 min. The absorbance was recorded at 510 nm, and a standard curve was generated with rutin concentration on the x-axis and absorbance on the y-axis. for sample analysis, 0.4 mL of the sample extract was processed using the same procedure as the standard preparation. The total flavonoid content was calculated based on the standard curve and expressed as milligrams of rutin equivalents per gram of sample (mg RE/g).

### 2.5. Determination of Caffeine, Trigonelline, and Chlorogenic Acid

Caffeine was determined using the national standard “National Food Safety Standards: Determination of Caffeine in Beverages” (GB 5009.139—2014) [39] with minor modifications. Briefly, 1.0 g of the dried sample was mixed with 200 mL of distilled water and extracted in a boiling water bath for 30 min. Upon cooling, 5 g of MgO was added, and the mixture was homogenized, followed by further extraction in a boiling water bath for 20 min. Finally, the solution was diluted to 250 mL with distilled water and allowed to stand for a while. The supernatants were filtered through a 0.22 µm syringe filter for analysis. Caffeine standard solutions with different concentrations were prepared for the calibration curve. The caffeine contents were quantified using an ultra-high-performance liquid chromatograph (UHPLC) (Thermo Fisher Scientific, Vanquish Flex, Shanghai, China) equipped with a C18 column (250 × 4.6 mm, 5 µm) and UV detector. An isocratic mobile phase consisted of solvent A (24% methanol) and solvent B (76% water) at a flow rate of 0.3 mL/min. Column temperature was set at 30 °C, and an injection volume of 1 µL was analyzed at 272 nm.

The content of trigonelline was determined by referring to the method reported in the literature, with appropriate modifications [40]. Briefly, 0.5 g of the dried sample was extracted with 80 mL of boiling water for 30 min. Once cooled to room temperature, 1 mL of 5% sulfosalicylic acid solution was added, mixed well, and the volume was brought up to 100 mL with distilled water. The supernatant was filtered through a 0.22 µm microporous membrane for analysis. High-performance liquid chromatography (HPLC) was conducted using an amino column (4.6 mm × 250 mm) with the following settings: detection at 260 nm, a column temperature of 30 °C, an injection volume of 10 µL, and an isocratic elution with mobile phase composed of 88% methanol (A) and 12% water (B) at a flow rate of 0.80 mL/min.

The methods for chlorogenic acid and its derivatives were adopted from a previously reported method [41]. To extract chlorogenic acid, 0.5 g of the dried sample was placed in a 50 mL centrifuge tube and mixed with 30 mL of 50% methanol. The mixture was ultrasonically extracted for 30 min., diluted to the mark with 50% methanol, centrifuged, and filtered through a 0.22 µm membrane for analysis. Standard curves were generated for chlorogenic acid (CAS: 327-97-9, Beijing Solarbio Science & Technology Co., Ltd., Beijing, China), neochlorogenic acid (CAS: 906-33-2, Beijing Solarbio Science & Technology Co., Ltd., Beijing, China), cryptochlorogenic acid (CAS: 905-99-7, Beijing Solarbio Science & Technology Co., Ltd., Beijing, China), and 3,5-di-O-caffeoylquinic acid (CAS: 89919-62-0, Beijing Solarbio Science & Technology Co., Ltd., Beijing, China). The analysis was performed using an ultra-high-performance liquid chromatography equipped with triple quadrupole mass spectrometer (UHPLC–MS/MS, Thermo Fisher Scientific, TSQ Quantis, Shanghai, China). A C18 column (5 µm, 4.6 × 250 mm) was used, with mobile phase solvents A (acetonitrile) and B (0.1% formic acid). The gradient elution program was as follows: 0–1 min, 10% A + 90% B; 2–4 min, 40% A + 60% B; 5–6 min, 80% A + 20% B; and 6–6.5 min, 10% A + 90% B. The flow rate was maintained at 0.1 mL/min, the column temperature at 30 °C, and the injection volume at 2 µL. Mass spectrometry conditions were as follows: electron spray ion source, multi-reaction monitoring mode, negative ion mode, spray voltage 4500 V, ion source temperature 550 °C, gas curtain gas pressure 206 kPa, and atomization gas/auxiliary gas pressure 344 kPa. Other parameters are shown in Table 1.

### 2.6. Sensory Profiling

An electronic nose system (AIRSENSE PEN3+EDU3, AIRSENSE Analysetechnik GmbH, Schwerin, Germany) was used to identify the sensory characteristics of volatile compounds in green coffee beans (Oe+Ie) and coffee byproducts (Ep, Pc, Sk). The device consisted of 10 chemical sensors, each designed to detect specific classes of compounds as listed in Supplementary Table S1. A 2.0 g sample of each dried tissue was placed in a 20 mL glass vial sealed with a silicone cap and allowed to equilibrate for 30 min before headspace sampling. The measurement time was set to 60 s, with a carrier gas flow rate of 400 mL/min and a cleaning time of 60 s between samples. Each sample was analyzed in five replicates to ensure the reliability of the test. For taste analysis, an electronic tongue system (INSENT SA-402B, Intelligent Sensor Technology, Inc., Japan) was used to assess the taste profiles of green coffee beans (Oe+Ie) and byproducts (Ep, Pc, Sk). The system was equipped with seven sensors and an automatic sampler, allowing the detection of sweetness, sourness, astringency, bitterness, saltiness, and umami. Each sample was analyzed for 30 s, and all measurements were conducted in five replicates.

### 2.7. Determination of Volatile Compounds

#### 2.7.1. HS-SPME: Sampling and Parametric Conditions

According to the methods of Zhai et al. [42] with slight modifications, volatile compounds were extracted using headspace solid-phase microextraction (HS-SPME) (CTC Analytics AG, PAL3, Switzerland). Briefly, 1.0 g of dried powder was placed in a 20 mL headspace vial sealed with a screw cap and a polytetrafluoroethylene (PTFE)/silicone septum. Then, 10.0 µL of 3-heptanone internal standard solution (1 µg/mL) was added 1 h before GC–MS analysis. The vial was stirred at 350 r/min for 40 min at 60 °C. A C-WR/PDMS/10 extraction fiber (95 µm) was used to adsorb volatile compounds (C-WR/PDMS is a hybrid coating in which Carbon Wide Range enhances the adsorption capacity for polar compounds while maintaining high sensitivity to non-polar compounds. PDMS exhibits good adsorption performance for non-polar or weakly polar volatile compounds, such as alkanes and aromatic hydrocarbons. C-WR/PDMS has been demonstrated to enable efficient and comprehensive extraction of a broad spectrum of volatile organic compounds from wine [43], and food contact paperboard [44]). The fiber was then thermally desorbed at 250 °C for 3 min, and the sample was immediately injected into the GC system.

#### 2.7.2. GC-MS Instrumentation and Conditions of Analysis

Referring to a previously reported laboratory method [45], volatile compounds were analyzed using an Agilent 7890A gas chromatograph coupled to a 5975C quadrupole mass spectrometer (Agilent Technologies Inc., USA). A polar HP-INNOWAX capillary column (60 m × 0.25 mm × 0.25 µm; Agilent, Santa Clara, CA, USA) was used. Electron ionization (EI) energy was set to 70 eV. The oven was initially set to 50 °C and maintained at that temperature for one minute. Then, it was gradually heated to 220 °C at a rate of 3 °C per minute, after which it was held at 220 °C for five minutes. The injector temperature was 250 °C, with helium as the carrier gas at a flow rate of 1.0 mL/min and a split ratio of 5:1. The quadrupole and ion source temperatures were set at 150 °C and 230 °C, respectively, with a total runtime of 62.66 min. For mass spectrometric detection electron ionization (EI) mode was used with an ionization energy of 70 eV. The ion source temperature was 230 °C, the transfer line was maintained at 250 °C, and the quadrupole at 150 °C. Mass spectra were collected in scan mode over the m/z range of 30–350, while a standard mixture of saturated hydrocarbons (C_7_–C_30_) was analyzed under the same chromatographic conditions to determine retention indices (RIs).

#### 2.7.3. Identification of Volatile Compounds and Semi-Quantification

The NIST 11.0 mass spectral library (National Institute of Standards and Technology) was used to identify volatile compounds. For statistical analysis, only volatiles identified in replicated samples with a library match probability >80% were included. Linear retention index (RI) values were calculated using the retention times of an n-alkane mixture (C7–C30). The calculated RI was compared with the RI in the NIST Chemistry WebBook and the RI in the literature (measured on a stationary phase chromatographic column of the same type as in this study, namely HP-INNOWAX). When the RI value of a compound deviates from the literature value within ± 20 units, the identity of the compound is confirmed. The confidence of identification was reported following the Metabolomics Standards Initiative (MSI) levels [46], with most assignments corresponding to Level 2 (putative annotation). Results were reported as semi-quantitative concentrations (C, µg/g) relative to the internal standard (IS) 3-heptanone.

(3) $$C(\mu g/g)={(Area}_{Volatile\;compound}/{Area}_{IS})\times C_{IS}$$

### 2.8. Statistical Analysis

One-way analysis of variance (ANOVA) was performed in Origin 2021, and Duncan’s test was used to compare means at the 5% significance level (*p* < 0.05). Data are presented as mean ± standard deviation (SD). Flavor radar plots were generated in Origin 2021. Clustering heat maps, Venn diagrams, and PCA were generated using Metware Cloud (https://cloud.metware.cn, accessed on 5 October 2024). Heat maps were Z-score standardized, with color gradients indicating relatively high and low values. PCA is a linear dimensionality reduction method that projects high-dimensional data into a low-dimensional space for visualization [47]. PCA was performed in R (version 3.5.1) using UV scaling. Venn diagrams were used to compare relationships among sets and to identify shared or specific metabolites among samples. Each dataset was measured at least three times.

## 3. Results and Discussion

### 3.1. Assessing Fresh Weight and Moisture Content

Figure S1 displays the fresh weight ratios and moisture content of seven different coffee varieties’ green beans (Oe, Ie) and their tissue parts (Ep, Pc, and Sk). As shown in Figure S1a, the fresh weight ratio of coffee green beans ranges from approximately 24% to 41%, with RY3, RY5, DR390, and DR402 exhibiting higher ratios (41.40%, 35.42%, 35.15%, and 32.49%, respectively). The combined fresh weight proportions of byproducts (Ep, Pc, and Sk) ranged from 59% to 76%, with Ep accounting for the largest share (35–50%) and Sk the smallest (1.4–3.0%). Literature reports indicate that coffee husk (including Ep and Pc) constitutes the primary byproduct of coffee fruit, making up about 45% of the dry weight [48]. Consistent with previous studies, Ep and Pc showed high proportions in this study.

Moisture content data provide a basis for drying and storing green coffee beans (Oe+Ie) and byproducts (Ep, Pc, Sk). As shown in Figure S1b, the moisture content of different green coffee beans (Oe+Ie) ranged from 30% to 45% and from 26% to 45%, respectively, indicating minimal variation between Oe and Ie. Among the byproducts, Ep exhibited the highest moisture content (55–74%), while Sk had the lowest (6–12%), showing a significant difference. Previous studies reported Sk moisture content at approximately 5–10% [49], which aligns closely with our experimental results. Given the substantial moisture variation across different coffee parts, drying times should be tailored to their specific moisture levels during practical drying processes. Overall, Ep appeared to be the main by-product. Sk, due to its very low moisture content, has more stable physicochemical properties.

### 3.2. Assessment of Antioxidant Potential of Coffee Fruit Structural Tissues

ABTS+• and DPPH• scavenging capacities, along with ferric reducing antioxidant power (FRAP), are commonly used indicators for evaluating antioxidant activity. Studies have shown that coffee byproducts demonstrate strong ABTS+•, DPPH• scavenging activity and FRAP [50]. Table 2 indicates that the ABTS+• scavenging rates for coffee Ep, Oe, and Ie all exceeded 85%, which is significantly higher than those for Pc and Sk (*p* < 0.05). However, differences between varieties were negligible. The DPPH• scavenging rates of Ep, Oe, and Ie all exceeded 90%, significantly higher than those of Pc and Sk (*p* < 0.05). FRAP assay results showed a similar trend, with Ep, Oe, and Ie exhibiting stronger FRAP activity than Pc and Sk. These findings suggest a certain correlation among ABTS+•, DPPH• scavenging capacity, and FRAP. Previous studies have reported that coffee Sk exhibits high antioxidant activity, with DPPH• and FRAP antioxidant values ranging from 206 to 287 μmol Trolox equivalents/g and 95 to 217 μmol Fe^2+^/g, respectively [50].

There were no significant differences in ABTS+• scavenging rates among coffee varieties Ep, Oe, and Ie (*p* > 0.05). For Pc and Sk, DR397 exhibited the lowest ABTS+• scavenging rate (*p* < 0.05), while no significant differences were found among the other varieties (*p* > 0.05). For Ep, the DPPH• scavenging capacity was higher in Arabica coffee than in Robusta coffee. In contrast, for green coffee beans (Oe+Ie), the DPPH• scavenging capacity was higher in Robusta coffee than in Arabica coffee. No clear pattern was observed for FRAP among different coffee varieties.

In summary, both green coffee beans (Oe+Ie) and Ep exhibit high antioxidant activity. Among them, the coffee beans (Oe+Ie) consistently showed the highest antioxidant capacity among all examined tissues (Ep, Pc, Sk, Oe+Ie). Bioactive compound (polyphenols and flavonoids) contents have been shown to strongly correlate with antioxidant capacity [51]. Subsequent analyses would also indicate that green coffee beans (Oe+Ie) contain higher levels of polyphenols and flavonoids. The byproducts (Ep, Pc, and Sk) exert strong antioxidant activity, indicating they could be valuable sources of natural antioxidants [52]. Additionally, studies have investigated the use of coffee husk extracts as antioxidants to slow down lipid oxidation, demonstrating promising potential for practical applications [53].

### 3.3. Determination of Soluble Sugars, Total Phenolic, and Flavonoid Contents

**Figure 2:**
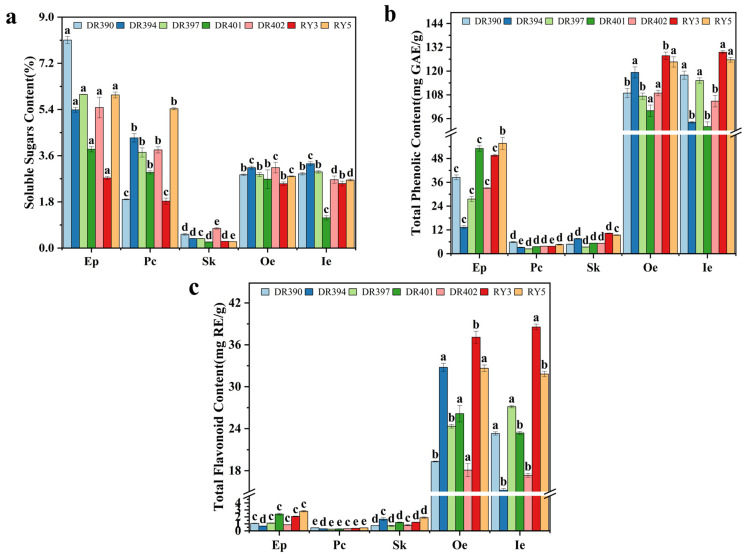
Differences in soluble sugar (**a**), total phenolic content (TPC) (**b**), and total flavonoid content (TFC) (**c**) among different tissues of coffee fruit of different varieties. Gallic Acid Equivalents (GAE), Rutin Equivalents (RE). exocarp (Ep), parchment (Pc), silverskin (Sk), outer endosperm (Oe), and inner endosperm. De’re 390 (DR390), De’re 394 (DR394), De’re 397 (DR397), De’re 401 (DR401), De’re 402 (DR402), Reyan No.3 (RY3), Reyan No.5 (RY5). Different letters above the bars indicate statistically significant differences (*p* < 0.05).

Figure 2 displays the soluble sugar, TPC, and TFC in various tissue regions of coffee cherries (Ep, Pc, Sk, Oe, Ie). Sugars are key precursors for the development of coffee flavor and aroma [54]. During coffee roasting, sugars react with amino acids, peptides, and proteins through Maillard reactions, giving coffee its unique flavor profile. As depicted in Figure 2a, the soluble sugar content in green coffee beans (Oe+Ie) ranged from 3.86% to 6.42%, lower than the values reported in some literature [54], potentially due to differences in coffee variety and maturity. Among green coffee beans (Oe+Ie), DR394 (Arabica) and RY5 (Robusta) had relatively high soluble sugar contents. Among the byproducts, Ep showed significantly higher soluble sugar content (*p* < 0.05), ranging from 2.73% to 8.10%, although it was lower than the 14.6% reported by Hu et al. [55]. Among the varieties, the Ep soluble sugar content of DR390 was the highest. Pc contained soluble sugars at 1.83–5.43%. The higher sugar content in Ep and Pc may be due to their pectin richness, as pectin degrades into soluble sugars during fruit ripening [56].

As shown in Figure 2b, among various parts, green coffee beans (Oe+Ie) exhibited significantly higher TPC than byproducts (*p* < 0.05), ranging from 91.94 to 129.47 mg GAE/g. This finding aligns with the results reported by Machado et al. [33]. At varietal level, DR390 (Arabica) and RY3 (Robusta) showed relatively high TPC. In DR394, DR401, DR402, and RY5, Oe showed a higher TPC than Ie, while the opposite was true for DR390, DR397, and RY3, indicating variations in phenolic compound accumulation among the cultivars. The TPC (13.19–55.24 mg GAE/g) of coffee Ep exceeded the value (6.08 mg GAE/g) reported by Hu et al. [55], possibly due to the reduced oxidative loss of polyphenols during low-temperature drying in this study [57,58]. In addition, TPC variations among samples also correlate with cultivar, climate, and cultivation conditions [59]. Other studies have reported TPC values of 23.70, 1.80, and 12.80 mg GAE/g for coffee Ep, Pc, and Sk, respectively [33]. In this study, the TPC of Ep was comparable to these reported values, whereas that of Pc was lower. Discrepancies in TPC values among studies may stem from differences in quantitative methods, extraction techniques, and sample origins [60]. The TPC of coffee Sk ranged from 3.36 to 10.27 mg GAE/g, lower than values (8–32 mg GAE/g and 20 mg GAE/g) reported in the literature [49,61]. Furthermore, the TPC of Pc ranged from 2.42–5.84 mg GAE/g, which aligns with the results reported in literature (2.28–2.84 mg GAE/g) [62]. Although the TPC of coffee Ep (13.19–55.24 mg GAE/g) was lower than that of green coffee beans (Oe+Ie), it was higher than values reported for apple peel (3.2 mg GAE/g) [63], myrtle fruit peel (14.06 mg GAE/g) [64] and passion fruit peel (35.95 mg GAE/g) [65]. This suggests that coffee husks are a potential source of polyphenolic compounds and could be used to develop functional products.

As shown in Figure 2c, the TFC in green coffee beans (Oe+Ie) (15.19–38.55 mg RE/g) was significantly higher than in byproducts (Ep, Pc, Sk) (*p* < 0.05). Among coffee beans, DR397 (Arabica) and RY3 (Robusta) showed relatively high TFC. Specifically, the TFC in the Oe and Ie of DR394, DR401, DR402, and RY5 showed higher TFC levels in Oe, consistent with the TPC trend. This indicates that TPC and TFC in these four coffee varieties are mainly concentrated in the outer layers of the coffee bean. Among byproducts, Ep showed relatively high TFC (0.67–2.80 mg RE/g), although lower than values reported in other studies [55].

Among different coffee varieties, DR390 had higher soluble sugar content in Ep, while RY5 had higher soluble sugar content in Pc. The Robusta coffee (RY series) of green coffee beans (Oe+Ie) contained higher TPC and TFC than the Arabica coffee (DR series). In the DR and RY series, the varieties with the highest TPC in green coffee beans (Oe+Ie) were DR390 and RY3, respectively; RY3 green coffee beans (Oe+Ie) also had higher TFC. In conclusion, among tissues, soluble sugar contents in Ep and Pc were higher than in other tissues, whereas TPC and TFC in green coffee beans were significantly higher than in byproducts. Among varieties, Robusta coffee had higher TPC and TFC than Arabica, with RY3 beans showing the highest values.

### 3.4. Determination of Caffeine, Trigonelline, and Chlorogenic Acid Contents

Caffeine, trigonelline, and chlorogenic acid are the key metabolites that influence coffee quality and flavor. Among these, caffeine and trigonelline are important alkaloids, both linked to coffee bitterness [66,67]. As shown in Table 3, caffeine levels vary significantly across different tissues. For Arabica coffee (DR series), the caffeine content across tissues followed Oe and Ie > Ep > Sk > Pc, while in Robusta coffee (RY series), the order was Oe and Ie > Sk > Ep > Pc. Caffeine content in DR series beans ranged from 1.0% to 1.3%, consistent with the range reported by Costa et al. [50]. Among green coffee beans (Oe+Ie), DR390 and DR402 showed relatively high caffeine contents, whereas DR397 had the lowest. The caffeine content of RY series green coffee beans (Oe+Ie) ranged from 2.0% to 2.6%, which is twice that of the DR series, aligning with findings from other studies [67,68]. RY3 green coffee beans had a higher caffeine content than RY5. Among different coffee byproducts (Ep, Pc, Sk) tissues, Ep exhibited higher caffeine content, slightly exceeding the measurements as reported by Hu et al. [55]. Similarly, Tran et al. also found higher contents of caffeine in Ep [69]. Previous studies have documented comparable caffeine content (6 mg/g) in coffee Sk [49], comparable to that of RY5’s Sk in our study. The caffeine levels (0.54–3.00 mg/g) in Pc align with values (1.3 mg/g) reported by Mirón-Mérida et al. [70].

The distribution of trigonelline varied among different coffee tissue types. In varieties DR390, DR394, DR397, and RY5, the concentration sequence was green coffee beans (Oe+Ie) > Ep > Pc > Sk, whereas in varieties DR401 and DR402, it followed the order green coffee beans (Oe+Ie) > Pc > Ep > Sk. This indicates that the trigonelline content in green coffee beans (Oe+Ie) was higher than in byproducts. Among them, DR390 (Arabica) and RY3 (Robusta) green beans (Oe+Ie) showed relatively high trigonelline contents. Among byproducts (Ep, Pc, Sk), trigonelline in Ep (2.58–6.06 mg/g) was higher than in Pc and Sk and was slightly higher than that reported by Hu et al. [55].

The content patterns of chlorogenic acid, neochlorogenic acid, cryptoclorogenic acid, and 3,5-di-O-caffeoylquinic acid in coffee beans were similar across various parts, with the order: green coffee beans (Oe+Ie) > Sk > EP > Pc. The four chlorogenic acids in Oe and Ie were significantly higher than those in Sk, Ep, and Pc (*p* < 0.05). For DR390, DR394, DR402, RY3, and RY5, chlorogenic acid, neochlorogenic acid, cryptoclorogenic acid, and 3,5-di-O-caffeoylquinic acid all exhibited Oe > Ie, suggesting that chlorogenic acid mainly accumulates in the outer layers of coffee beans. Additionally, the RY series exhibited higher chlorogenic acid content than the DR series, consistent with findings reported by Lemos et al. [67]. This rule also applies to the caffeine content. Among coffee byproducts (Ep, Pc, Sk), the content of chlorogenic acid and its derivatives in Sk was significantly higher than that in Ep and Pc, suggesting that Sk could be a key coffee byproduct for chlorogenic acid extraction. Ep and Pc contained chlorogenic acid at 0.59–1.28 mg/g and 0.092–0.126 mg/g, respectively, slightly lower than data reported in other studies [20]. Machado et al. [33] reported neochlorogenic acid contents of 2.20, 0.05, and 0.52 mg/g in coffee Ep, Pc, and Sk, respectively, differing from our findings.

In conclusion, this study shows that caffeine, trigonelline, and chlorogenic acid contents in green coffee beans (Oe+Ie) are significantly higher than in coffee byproducts (Ep, Pc, Sk). Consistently, coffee beans also showed higher total phenols, total flavonoids, and antioxidant activities (as measured by the ABTS and FRAP assays). These results suggest that the stronger antioxidant capacity of coffee beans may be attributed to their higher levels of bioactive components. Among varieties, Robusta (RY series) had higher caffeine and chlorogenic acid contents than Arabica (DR series). Coffee beans with relatively high caffeine contents were DR390, DR402, and RY3, and those with relatively high chlorogenic acid contents were DR397, DR402, and RY3.

### 3.5. Electronic Sensory Analysis

**Figure 3:**
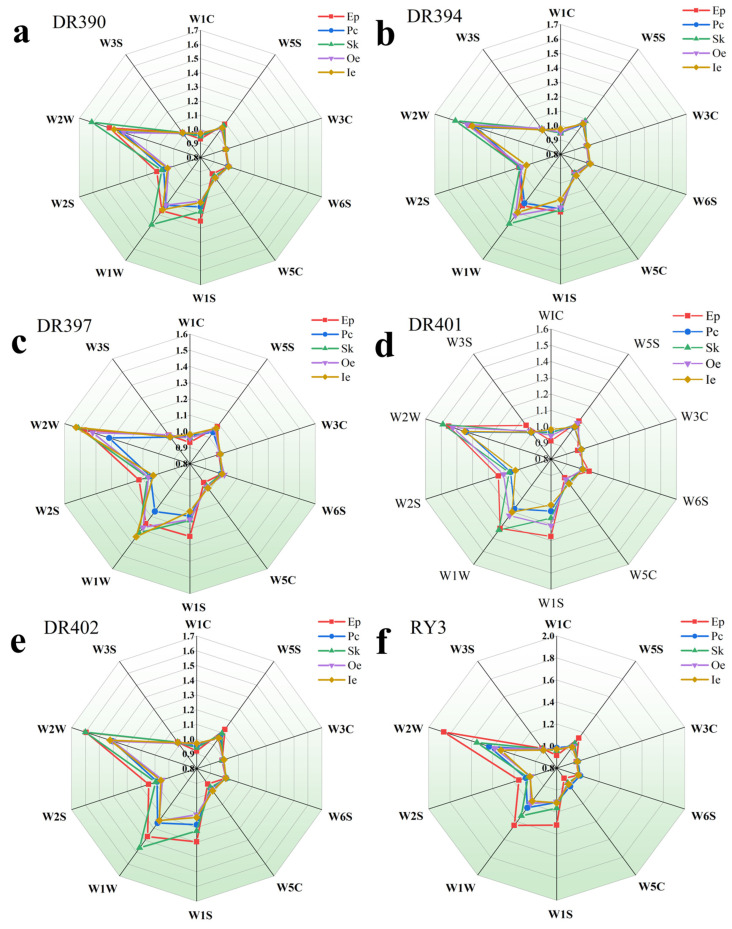

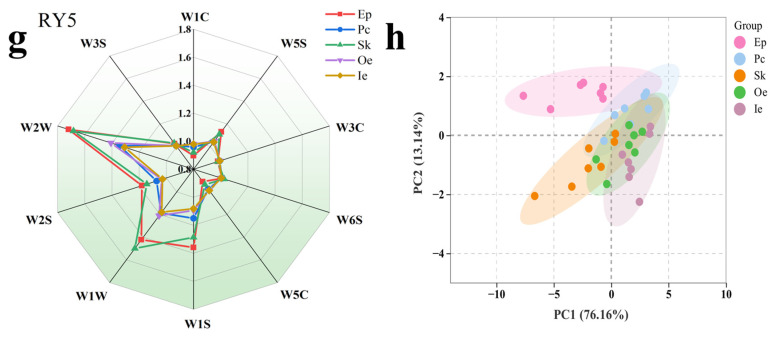
Electronic nose analysis of different coffee fruit tissues across varieties. Panels (**a**–**g**) show radar plots for DR390, DR394, DR397, DR401, DR402, RY3, and RY5, respectively. Panel (**h**) shows PCA of electronic nose responses. Tissues: exocarp (Ep), parchment (Pc), silverskin (Sk), outer endosperm (Oe), inner endosperm (Ie). Sensor selectivity: W1C (aromatics), W5S (nitrogen oxides), W3C (ammonia and aromatics), W6S (hydrogen), W5C (short-chain alkanes and aromatics), W1S (methane), W1W (terpenes and sulfides), W2S (alcohols, aldehydes, ketones), W2W (aromatics and organic sulfides), W3S (long-chain alkanes). Varieties: De’re 390 (DR390), De’re 394 (DR394), De’re 397 (DR397), De’re 401 (DR401), De’re 402 (DR402), Reyan No.3 (RY3), and Reyan No.5 (RY5).

As an objective bionic olfactory instrument, the electronic nose can rapidly and digitally capture and distinguish the overall volatile odor fingerprint of a sample. It is widely used for the rapid detection of volatile organic compound characteristics [71]. Figure 3a–g shows the response intensity of volatile organic compounds in different green coffee beans (Oe+Ie) and byproducts (Ep, Pc, Sk). Results show that responses to W2W and W1W were significantly higher across all tissue types compared to other sensors, indicating increased levels of aromatic compounds and sulfides in these samples. The response curves of Oe and Ie closely overlap, suggesting similar flavor profiles. Previous studies have shown that Arabica coffee generally possesses a higher aroma intensity than Robusta [72]. However, in the current study, this difference was not significant, possibly because the characteristic flavor distinctions between the two varieties had not yet developed in the unroasted coffee beans [73]. Among varieties, DR390 and DR397 (Arabica) and RY3 (Robusta) showed higher responses to the W2W sensor, indicating stronger aroma in these beans. In addition, Ep from DR402 and RY3 showed strong responses to W2W. All Pc response values were relatively weak, indicating overall low levels of volatile flavor compounds in Pc. PCA was conducted on the e-nose data to better understand the overall distribution patterns of volatile compounds in green coffee beans (Oe+Ie) and byproducts (Ep,Pc,Sk). As shown in Figure 3h, principal component 1 (PC1) and principal component 2 (PC2) together contributed over 80% of the variance, effectively representing the original data information. In the PCA score plot, Oe, Ie, and Pc cluster closely together, especially Oe and Ie, which shows they have similar flavor profiles. Ep, however, was distinctly separated from the other four tissues, suggesting differences in its volatile compound composition. Overall, different coffee tissues showed distinct responses to W2W and W1W, indicating that aroma-related compounds and sulfides are major contributors to flavor. DR390, DR397, and RY3 beans showed stronger W2W responses. The flavor profiles of Oe, Ie, and Pc were similar, whereas Ep differed markedly from Pc, Sk, Oe, and Ie.

**Figure 4:**
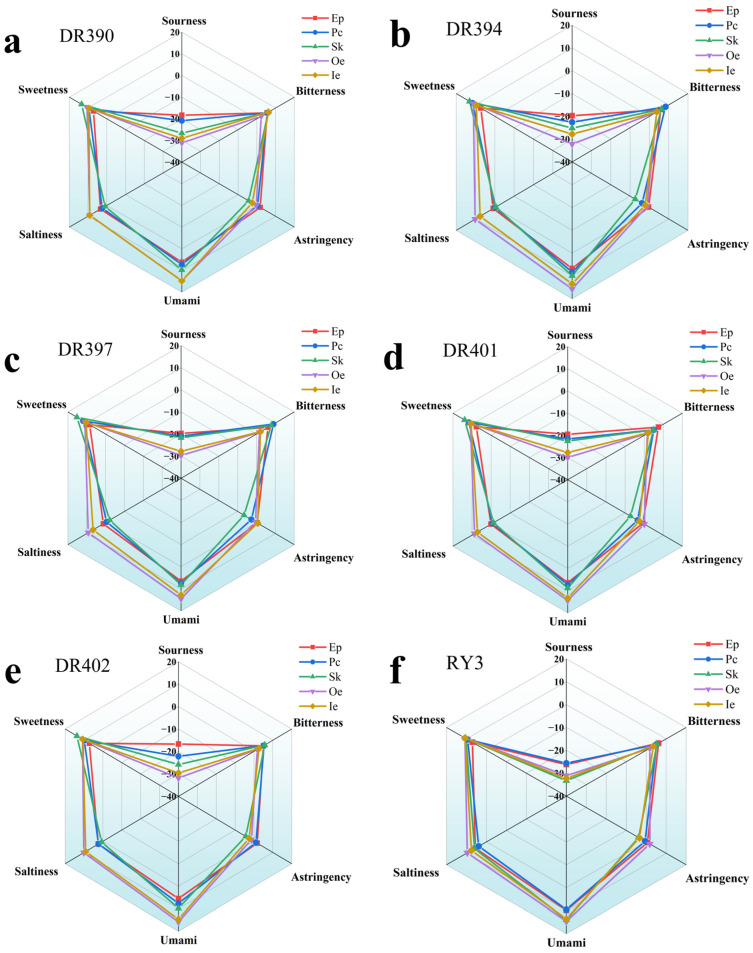

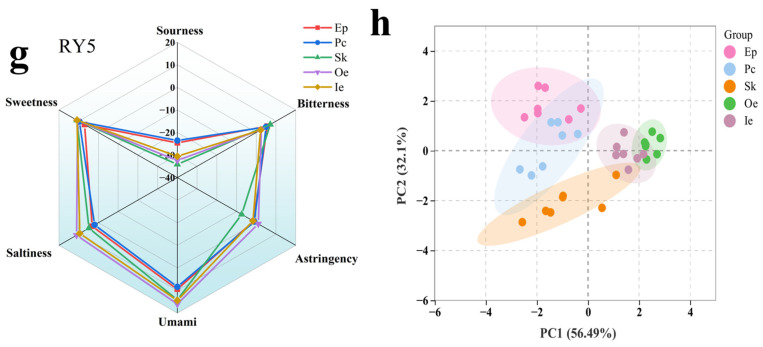
Electronic tongue analysis of different coffee fruit tissues across varieties. Panels (**a**–**g**) show electronic tongue results for DR390, DR394, DR397, DR401, DR402, RY3, and RY5, respectively. Panel (**h**) shows PCA of electronic tongue results. Tissues: exocarp (Ep), parchment (Pc), silverskin (Sk), outer endosperm (Oe), inner endosperm (Ie). Varieties: De’re 390 (DR390), De’re 394 (DR394), De’re 397 (DR397), De’re 401 (DR401), De’re 402 (DR402), Reyan No.3 (RY3), and Reyan No.5 (RY5).

The electronic tongue simulates human taste perception through a sensor array, enabling objective analysis and digital representation of taste characteristics. It holds significant applications in food quality control and flavor evaluation [74]. Figure 4a–g shows the electronic tongue detection results for the different tissues of coffee fruit from different varieties. Overall, all samples demonstrated low acidity but higher responses for umami, sweetness, saltiness and bitterness. Similarly. Flambeau et al. [75] measured the taste characteristics of coffee and found the relatively high response for sweetness and bitterness. It might be attributed to the presence of flavor substances such as soluble sugar, caffeine, and chlorogenic acid. To further explore the overall taste relationships among samples, PCA was performed on the electronic tongue-generated data (Figure 4h). The results showed that Ep and Pc, as well as Oe and Ie, grouped together, indicating that Ep and Pc, and Oe and Ie share similar taste traits. This similarity was also evident in the flavor radar of the electronic tongue chart.

In summary, the flavors of green coffee beans (Oe+Ie) and byproducts (Ep, Pc, Sk) are primarily dominated by aromatic compounds and sulfides, presenting low acidity, high sweetness, bitterness and umami. Among these, Oe and Ie exhibit relatively consistent flavor and taste profiles. Although Ep differed from Pc, Sk, Oe, and Ie in terms of flavor characteristics, its taste profile was similar to that of Pc. Among the green coffee beans (Oe+Ie) and Ep, the Arabica coffee with the higher aromatic components included varieties DR397 and DR402, while the Robusta coffee varieties with a relatively high content are represented by RY3. The intensity of taste contours among different samples was relatively similar, making it challenging to distinguish the taste characteristics among samples [75]. Therefore, further research is needed.

### 3.6. Analysis of Volatile Compounds in Different Coffee Green Beans and Byproducts

**Figure 5:**
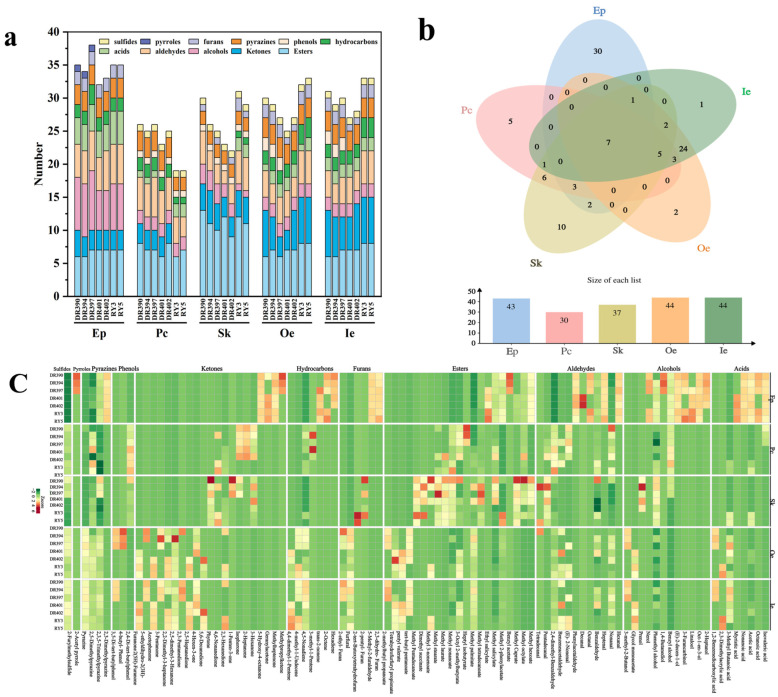
Accumulation of volatile compounds among the different tissues of coffee fruit of different varieties: (**a**) Stacked chart showing the number of volatile compounds in various tissues; (**b**) Venn diagram illustrating shared volatile compounds across tissues and (**c**) Cluster heatmap of volatile compounds in different tissues. exocarp (Ep), parchment (Pc), silverskin (Sk), outer endosperm (Oe), inner endosperm (Ie). Varieties De’re 390 (DR390), De’re 394 (DR394), De’re 397(DR397), De’re 401 (DR401), De’re 402 (DR402), Reyan No.3 (RY3), Reyan No.5 (RY5).

Volatile compounds in seven different green coffee beans (Oe+Ie) and their byproducts (Ep, Pc, Sk) were analyzed using HS-SPME-GC-MS. Compound identification was based on matching against the NIST 11.0 standard library and verified by consulting relevant literature [36,37,38]. A total of 11 classes of volatile compounds were identified, including esters, ketones, alcohols, aldehydes, acids, hydrocarbons, phenols, pyrazines, furans, pyrroles, and sulfur-containing compounds (Figure 5a,b). These compound categories correspond with those found in the literature [76,77,78].

The clustering heat map (Figure 5c) shows relative changes in the contents of 96 volatile compounds across tissues and varieties. The Venn diagram (Figure 5b) further highlights differences in volatile components among different tissues. Ep, Pc, Sk, Oe, and Ie each possessed 30, 5, 10, 2, and 1 unique volatile compounds, respectively, indicating that the volatile profile of Ep differs significantly from the others. Oe and Ie share 42 common volatile compounds, demonstrating very consistent aromatic features. Five different coffee tissues shared seven volatile compounds: methyl salicylate, phenethyl alcohol, nonanal, benzaldehyde, acetic acid, 2,3,5-trimethylpyrazine, and 2,5-dimethylpyrazine (Supplementary Table S2). These compounds collectively impart flavor characteristics such as bitter almond notes, fruity aromas (like cherry, apple, and lemon), floral scents, and caramel, malt, cocoa, and popcorn to different coffee tissues.

Out of the 43 volatile compounds identified in coffee exocarp (Ep), esters, alcohols, and aldehydes were the most common, playing a key role in creating the distinctive floral and fruity flavor aromas [79]. Twenty-two shared compounds were found across different coffee Ep samples (Supplementary Table S2-1). Among these, methyl salicylate, 5-hydroxy-4-octanone, oct-1-en-3-ol, hexanal, heptenal, 2,3,5-trimethylpyrazine, and 5-methyl-2-furaldehyde showed higher concentrations and are assumed to be key volatile compounds in coffee Ep. Previous studies have identified volatile compounds in coffee fruit Ep, such as hexanal, heptenal, phenylacetaldehyde, benzyl alcohol, phenethyl alcohol, and methyl palmitate, which are consistent with the findings of this study [55,80]. Alcohols in Ep are more abundant than those in other tissues. Likely due to higher alcohol dehydrogenase activity in mature fruit peel, which catalyzes the reduction of aldehydes and ketones into alcohols [81]. The accumulation of 5-methyl-2-furaldehyde and 2-acetyl pyrrole detected in coffee fruit Ep may have resulted from glycoside bonding during the drying process of the fruit peel [76]. Consistent with the electronic nose results, coffee Ep showed a strong response to the sensor for aromatic compounds. This may be attributed to the fruity and floral substances detected in coffee exocarp (Table S2-1), such as methyl hexoate, methyl caprate, benzyl acetate, (E)-2-decen-1-ol, phenethyl alcohol and octanal, etc.

Coffee parchment (Pc) yielded 30 volatile components, mostly esters and aldehydes, and among them, 15 were shared across different varieties. These include five esters (methyl octylate, methyl salicylate, methyl tetradecanoate, etc.), which give off fruity, wine-like, and honey aromas; two alcohols (benzyl alcohol, phenethyl alcohol) that are typical floral compounds; three aldehydes (nonanal, benzaldehyde, 2,4-dimethyl-benzaldehyde) contributing flavor notes such as fatty, floral, bitter almond, and fruity qualities.

Coffee silverskin (Sk) contained 37 components, with esters, ketones, and aldehydes accounting for a high proportion. Among the 18 volatile compounds common to the Sk of different varieties, those with relatively higher concentrations include methyl nonanoate, methyl tetradecanoate, methyl palmitate, 2,4-dimethylbenzaldehyde, phenylethyl alcohol, and 2,4-di-tert-butylphenol. Martínez et al. [71] identified 40 flavor compounds in coffee Sk and detected the same compounds as in the present study, including 2,3,5-trimethylpyrazine, acetic acid, and phenylethanol. The analysis of the electronic nose also revealed a strong response to coffee Sk aromatic components. which might be attributed to substances such as methyl hexoate, 3-hexanone, methyl salicylate, etc. (Table S2-3).

Green coffee beans (Oe+Ie) contained 44 volatile compounds, which were significantly fewer than the number in roasted coffee beans [82]. The composition was dominated by esters and ketones, followed by aldehydes. Esters exhibit high volatility and low detection thresholds, making them easily perceptible to humans and potentially significant contributors to the flavor of coffee beans [57], giving the coffee a fruity aroma [76]. The results of the electronic nose also showed a strong response to aromatic substances. Previous studies have shown that high-quality green coffee beans (Oe+Ie) contain higher levels of volatile esters and ketones, exhibiting typical floral aromas and other flavors. Low-quality coffee flavors such as pyridine, octanoic acid, and dimethyl sulfide [1] were not detected in green coffee beans. Aldehydes, generated via the ester oxygenase pathway (e.g., hexanal), impart fruity and grassy aromas [83]. Furan compounds are higher in green coffee beans (Oe+Ie) and Ep, possibly due to the breakdown of sugars and amino acids [84]. Among the seven green coffee bean (Oe+Ie) samples, 15 volatile compounds were shared, with higher relative contents of methyl palmitate, 2,4-dimethylbenzaldehyde, pyrazine, and 2,3,5-trimethylpyrazine. Although sulfur-containing compounds are relatively few, their odor thresholds are low [1], and they play a crucial role in coffee flavor formation. In this study, 2-furylmethyl sulfide, known for its roasted and caramel-like aromas, was identified as a key contributor to coffee’s characteristic flavor [85]. Pyrazine compounds, which impart strong nutty and spicy notes [59], along with the abundant presence of pyrazine and 2,3,5-trimethylpyrazine, further enhanced the flavor complexity of the green coffee beans (Oe+Ie). However, few studies have indicated that pyrazine compounds can diminish the quality of coffee flavor by imparting earthy notes [86]. Aldehydes are among the major volatile constituents [87], including nonanal, benzaldehyde, (E)-2-nonenal, and benzeneacetaldehyde. Furan derivatives such as furfural and 2-ethylfuran detected in the green coffee beans are likely formed during drying through acid-catalyzed degradation of Maillard reaction intermediates involving reducing sugars and amino acids [88]. Their presence in green coffee beans (Oe+Ie) has been linked with the reduced cup quality [1]. In addition, aldehydes and furans are known to possess roasted and smoky characteristics and are detected across different coffee varieties [89]. However, several volatile compounds reported in other studies—such as 2,3-diethyl-5-methylpyrazine, 2-isobutyl-3-methoxypyrazine, 2-/3-methylbutanoic acid, and phenylacetic acid [76,90], were not detected in this study. This could be attributed to the possibility of differences in coffee varieties or extraction methods for volatile compounds. Given the low volatile content of green coffee beans, Lee et al. [91] modified coffee aroma by fermenting green beans with *Rhizopus oligosporus*. Fermentation increased total volatiles by 36%, and degradation of ferulic and caffeic acids doubled the total concentration of volatile phenolic derivatives. Fermentation with *Lactobacillus rhamnosus* has also been used to adjust green coffee flavor; after fermentation, caramel notes were enhanced, whereas nutty notes were weaker [92]. Roasting disrupts the cellular structure of raw beans and releases bound aromatic compounds. With continued heating, caramelization occurs, and aldehydes, ketones, ethers, and other compounds volatilize, while grinding further releases aroma [93]. Flavor precursors in green beans transform during roasting to generate many new aroma compounds, giving roasted coffee richer and more complex flavor. Bhumiratana reported that all flavor compounds in green beans are also present in roasted beans, with additional aroma components formed after roasting [93]. In contrast, de Melo Pereira et al. [2] reported that volatiles in green beans, such as alcohols, aldehydes, and alkanes, have limited impact on final aroma, which mainly arises from Maillard reactions and caramelization during roasting. Although green-bean volatiles may not directly determine final aroma, their profiles can indicate variety, origin, and processing, and provide a baseline for analyzing flavor formation during roasting.

Among the volatile compounds in coffee, fruity, herbal, sweet, nutty, and spicy notes are seen as desirable flavors, while roasted, smoky, fermented, and earthy notes are considered undesirable [1]. We constructed flavor wheels to visualize the volatile profiles of different coffee bean (Oe+Ie) and byproducts (Ep, Pc, Sk). Ep primarily exhibited nutty, fruity, and floral aromas (Figure S2a); Pc showed dominant nutty and fruity notes (Figure S2b); Sk (Figure S2c) presented nutty and fruity characteristics accompanied by tobacco and wine-like nuances. Oe (Figure S2d) and Ie (Figure S2e) displayed similar profiles, with prominent nutty and floral notes, followed by fruity and waxy aromas. Consistent with the findings of Cascos et al. [1] fruity attributes were most pronounced in high-quality coffee.

### 3.7. Relative Odor Activity Values of Volatile Compounds

Flavor wheels are based on volatile compounds in coffee tissues to depict flavor, but their contribution depends on concentration and odor activity value [94], which is defined as the ratio of a substance concentration to its sensory threshold. A higher relative odor activity value (rOAV) indicates a greater contribution to the overall flavor profile. When rOAV exceeds 1, the compound is generally regarded as a key flavor compound [66].

Based on the aroma thresholds of 35 volatile compounds reported in the literature [42,95,96,97,98], this study calculated the rOAV for each compound. Among the 13 volatile compounds in green coffee beans (Oe+Ie) (Table 4), only four compounds exhibited rOAV exceeding 1. These include methyl salicylate, (E)-2-Nonenal, 2,3,5-trimethylpyrazine, and 2,5-dimethylpyrazine, which impart rich holly-like and fruity aromas. However, some studies have reported that green coffee beans (Oe+Ie) contain almost no pyrazines or other heterocyclic compounds; therefore, these substances are likely formed through chemical reactions occurring during the drying process [99]. Additionally, even though the odor activity values of Methyl 2-phenylacetate, Phenethyl alcohol, Nonanal, and Benzaldehyde were less than 1, they still notably influenced the flavor profile of green coffee beans (Oe+Ie). For instance, phenethyl alcohol is an important floral fragrance compound in coffee [1], while benzaldehyde has been detected in green coffee beans (Oe+Ie), contributing a characteristic bitter almond aroma [2]. These compounds collectively enhance the layered flavor complexity of green coffee beans (Oe+Ie).

Among the volatile compounds in various coffee byproducts (Ep, Pc, Sk) (Table 5), notable differences exist in key flavor compounds across the tissues. Ten volatile compounds in coffee Ep exhibited ROAVs exceeding 1, with Octanal and 2,3,5-Trimethylpyrazine showing rOAV > 100. These compounds significantly contribute to the aroma of coffee Ep, imparting pronounced fruity and cocoa notes. In coffee Pc, five compounds had rOAV > 1, while methyl salicylate, (E)-2-nonenal, and 2,5-dimethylpyrazine were the primary flavor contributors, imparting caramel notes, a dry, paper-like, nutty, and roasted aroma, respectively. In the Sk, key flavor compounds include methyl hexoate, methyl caprate, methyl 2-phenylacetate, methyl salicylate, 2,5-dimethylpyrazine, and 2-Pentylfuran. These components collectively impart fruity, sweet, and nutty flavors in Sk. Notably, methyl caprate exhibited the highest rOAV among different coffee silverskin, making it a key compound responsible for the fruity aroma.

In summary, relative odor activity values indicate that green coffee beans (Oe+Ie) primarily exhibit fruity, nutty, and floral aromas. Although waxes make up a significant part of the flavor wheel (Figure S2d,e), their compounds have relatively high sensory thresholds, thereby influencing the overall flavor profile only slightly. The coffee Ep contributed floral, fruity, and nutty notes. The flavor of Pc was dominated by nutty, roasted, and dry paper-like notes. The primary flavors of Sk were fruity and nutty. Although wine-like and tobacco notes were identified on the flavor wheel, their contribution to aroma remained to be further explored due to the lack of known odor thresholds for the corresponding compounds. Among different coffee varieties, the green beans of DR397, DR402, RY3, and RY5 exhibited pronounced flavors of fruit and nuts. Within the Ep group, DR390, DR397, DR402, and RY3 exhibited distinct floral, fruity, and nutty characteristics. A combined analysis of rOVA and e-sensory results indicates that DR397, DR402, and RY3 exhibited more pronounced overall fruity and nutty flavors across all varieties.

## 4. Conclusions

This study compared the differences in bioactive compounds, antioxidant capacity, and volatile flavor compounds among seven coffee varieties in different structural tissues. Regarding antioxidant properties, green coffee beans (Oe+Ie) and Ep showed the highest ABTS and DPPH scavenging abilities, along with the highest FRAP values. Green coffee beans also contained high levels of TPC, TFC, caffeine, trigonelline, and chlorogenic acid. Among the varieties, DR390 contained higher levels of total phenols, caffeine, and trigonelline, whereas DR402 was rich in caffeine and chlorogenic acid. Among Robusta coffees, RY3 showed higher TPC, TFC, caffeine, and chlorogenic acid. Among coffee byproducts, Ep appeared to be rich in soluble sugars, total phenols, caffeine, and trigonelline, Pc showed high soluble sugar content, while Sk exhibited rich chlorogenic acid contents. Among the varieties, DR390 contained higher levels of total phenols, caffeine, and trigonelline, while RY3 had elevated TFC and TPC, and DR397 and RY5 showed relatively higher chlorogenic acid content. In electronic sensory and GC-MS analyses, different coffee tissues primarily showed aromatic and sulfur compounds, with taste profiles characterized by low acidity and high sweetness. The Oe and Ie flavors were similar across tissues, while Ep showed notable variation. DR397, DR402, and RY3 exhibit pronounced aromatics, whereas DR397, DR402, and RY5 highlight more sweetness and umami, indicating distinct flavor profiles among these four varieties. Among coffee volatiles, esters were the most abundant. Seven compounds were shared across the coffee tissues, including methyl salicylate, phenethyl alcohol, and benzaldehyde, collectively contributing to the aromas of fruity, floral, and roasted notes. Ep exhibited fruity and cocoa notes, Pc presented a dry, papery aroma, Sk displayed fruity characteristics, while green coffee beans (Oe+Ie) revealed fruity and nutty aromas. Combined rOAV and e-sensory analysis indicated that DR397, DR402, and RY3 possessed more pronounced fruity and nutty flavors. In conclusion, this study demonstrates that green coffee beans (Oe+Ie) possess strong antioxidant properties and are rich in total phenols, total flavonoids, caffeine, and trigonelline. Based on tissue-specific traits, Ep may serve as a source of antioxidants and flavor enhancers, Pc is rich in sugars, and Sk shows high levels of chlorogenic acid. This suggests that coffee byproducts can be utilized for the development of high-value functional products. It can provide a scientific basis and material selection strategy for developing value-added products in the coffee industry chain, such as functional foods and antioxidants, thereby enhancing sustainability and economic returns. Regarding varieties, Arabica DR390 and DR402 and Robusta RY3 showed higher bioactive contents, stronger flavor characteristics, and better overall quality. Collectively, these findings provide a reference for promoting superior coffee varieties.

## Figures and Tables

**Table 1:** Mass Spectrometry Parameters of chlorogenic acid and its derivatives.

Compound	Retention Time (min)	Precursor Ion (*m*/*z*)	Product Ion (*m*/*z*)	Declustering Potential/V	Collision Energy/V
Chlorogenic acid	2.45	353	191	−100	−35
Neochlorogenic acid	1.61	354	178	−100	−30
Cryptochlorogenic acid	2.48	353	179	−100	−35
3,5-di-O-caffeoylquinic acid	2.90	515	173	−100	−30

**Table 2:** Antioxidant activity of different tissues in coffee fruit of different varieties.

Tissues	ABTS Scavenging Rate (%)						
	DR390	DR394	DR397	DR401	DR402	RY3	RY5
Ep	87.00 ± 0.13 ^aA^	85.69 ± 0.81 ^aAB^	85.79 ± 0.21 ^aAB^	85.90 ± 0.62 ^aAB^	85.29 ± 0.54 ^aB^	85.92 ± 0.14 ^aAB^	85.43 ± 0.47 ^aAB^
Pc	72.07 ± 0.10 ^cA^	71.60 ± 3.91 ^bA^	59.69 ± 1.45 ^bB^	75.10 ± 2.77 ^bA^	68.64 ± 1.19 ^cA^	70.89 ± 0.49 ^dA^	71.14 ± 1.76 ^bA^
Sk	79.68 ± 0.87 ^bA^	69.02 ± 0.94 ^bD^	59.50 ± 2.33 ^bE^	75.66 ± 2.90 ^bB^	71.68 ± 1.28 ^bCD^	75.81 ± 0.19 ^cB^	72.48 ± 0.81 ^bC^
Oe	85.57 ± 1.01 ^aAB^	85.32 ± 0.62 ^aAB^	86.41 ± 0.69 ^aA^	85.76 ± 0.31 ^aAB^	85.61 ± 0.64 ^aAB^	85.06 ± 0.79 ^bB^	85.74 ± 0.56 ^aAB^
Ie	86.18 ± 0.17 ^aA^	85.56 ± 0.62 ^aABC^	85.44 ± 0.65 ^aBC^	86.24 ± 1.40 ^aA^	85.96 ± 0.98 ^aAB^	85.22 ± 0.58 ^abC^	86.06 ± 0.28 ^aAB^
Tissues	DPPH scavenging rate (%)						
	DR390	DR394	DR397	DR401	DR402	RY3	RY5
Ep	95.27 ± 0.37 ^aA^	93.13 ± 0.86 ^aC^	95.02 ± 0.22 ^aA^	94.61 ± 0.95 ^aAB^	94.01 ± 0.55 ^aB^	92.44 ± 0.68 ^aCD^	91.77 ± 0.45 ^bD^
Pc	51.00 ± 2.80 ^cA^	33.36 ± 2.57 ^cCD^	30.00 ± 2.14 ^cDE^	28.86 ± 0.24 ^cE^	36.59 ± 2.96 ^cC^	45.32 ± 2.62 ^bB^	51.33 ± 0.36 ^bA^
Sk	32.67 ± 2.83 ^dCD^	38.37 ± 3.95 ^bB^	26.39 ± 2.15 ^dE^	28.81 ± 1.32 ^cDE^	31.24 ± 1.96 ^dBC^	39.86 ± 2.32 ^cB^	59.72 ± 1.20 ^cA^
Oe	91.02 ± 0.60 ^bE^	92.42 ± 0.78 ^aCD^	93.05 ± 0.84 ^abBC^	91.71 ± 0.36 ^bDE^	91.40 ± 0.67 ^abE^	93.68 ± 0.91 ^aAB^	94.18 ± 0.94 ^aA^
Ie	90.73 ± 0.58 ^bD^	92.56 ± 0.91 ^aB^	91.51 ± 1.52 ^bC^	91.81 ± 0.72 ^bC^	90.10 ± 0.39 ^bD^	92.77 ± 1.74 ^aA^	92.82 ± 1.72 ^abA^
Tissues	FRAP(µg FeSO4/mL)						
	DR390	DR394	DR397	DR401	DR402	RY3	RY5
Ep	8.15 ± 0.17 ^bB^	6.14 ± 0.02 ^cD^	8.26 ± 0.15 ^bB^	9.10 ± 0.26 ^bA^	7.56 ± 0.12 ^bC^	8.92 ± 0.11 ^cA^	8.95 ± 0.92 ^cA^
Pc	3.37 ± 0.02 ^cA^	2.41 ± 0.05 ^eD^	2.00 ± 0.03 ^cF^	2.34 ± 0.03 ^cE^	2.91 ± 0.02 ^cB^	2.77 ± 0.04 ^dC^	2.97 ± 0.03 ^eB^
Sk	2.18 ± 0.01 ^dE^	2.87 ± 0.05 ^dB^	1.98 ± 0.03 ^cF^	2.67 ± 0.03 ^cC^	2.25 ± 0.04 ^dE^	2.59 ± 0.02 ^dD^	3.33 ± 0.09 ^dA^
Oe	9.69 ± 0.10 ^aAB^	9.57 ± 0.03 ^bB^	9.81 ± 0.12 ^aAB^	9.72 ± 0.18 ^aAB^	9.63 ± 0.20 ^aAB^	9.84 ± 0.19 ^bA^	9.23 ± 0.06 ^bC^
Ie	9.58 ± 0.21 ^aBC^	9.71 ± 0.08 ^aBC^	9.82 ± 0.06 ^aB^	9.44 ± 0.27 ^aC^	9.68 ± 0.12 ^aBC^	10.17 ± 0.19 ^aA^	9.83 ± 0.06 ^aB^

Note: Different lowercase letters within the same column denote significant differences between tissues, whereas different uppercase letters within the same row signify significant differences between varieties (*p* < 0.05). exocarp (Ep), parchment (Pc), silverskin (Sk), outer endosperm (Oe), inner endosperm (Ie). De’re 390 (DR390), De’re 394 (DR394), De’re 397 (DR397), De’re 401 (DR401), De’re 402 (DR402), Reyan No.3 (RY3), Reyan No.5 (RY5).

**Table 3:** Differences in caffeine, trigonelline, and chlorogenic acid contents among the different tissues of coffee fruit of different varieties.

	Tissues	DR390	DR394	DR397	DR401	DR402	RY3	RY5
Caffeine (mg/g)	Ep	4.17 ± 0.02 ^bD^	5.68 ± 0.09 ^bC^	3.46 ± 0.04 ^cE^	7.49 ± 0.02 ^cB^	9.16 ± 0.03 ^cA^	2.47 ± 0.01 ^cF^	1.21 ± 0.02 ^dG^
	Pc	3.00 ± 0.01 ^cA^	1.10 ± 0.01 ^dC^	0.54 ± 0.03 ^eF^	0.88 ± 0.04 ^eD^	2.31 ± 0.00 ^eB^	0.54 ± 0.02 ^dF^	0.72 ± 0.10 ^eE^
	Sk	2.98 ± 0.03 ^cC^	4.47 ± 0.01 ^cB^	1.51 ± 0.02 ^dF^	1.89 ± 0.04 ^dD^	2.56 ± 0.01 ^dE^	3.01 ± 0.03 ^cC^	5.19 ± 0.01 ^cA^
	Oe	13.10 ± 0.47 ^aC^	11.98 ± 0.17 ^aD^	10.05 ± 0.05 ^bF^	10.84 ± 0.22 ^bE^	12.11 ± 0.11 ^bD^	23.24 ± 0.14 ^bA^	20.59 ± 0.13 ^bB^
	Ie	12.73 ± 0.30 ^aC^	12.08 ± 0.05 ^aD^	10.70 ± 0.09 ^aE^	11.57 ± 0.11 ^aD^	12.66 ± 0.10 ^aC^	26.01 ± 0.08 ^aA^	20.91 ± 0.12 ^aB^
Trigonelline (mg/g)	Ep	6.06 ± 0.03 ^cA^	5.59 ± 0.02 ^bB^	3.06 ± 0.03 ^cC^	2.67 ± 0.01 ^dD^	2.67 ± 0.02 ^dD^	2.98 ± 0.01 ^cC^	2.58 ± 0.01 ^cD^
	Pc	4.78 ± 0.03 ^dA^	2.62 ± 0.01 ^cC^	2.01 ± 0.01 ^dE^	3.05 ± 0.00 ^cB^	3.05 ± 0.01 ^cB^	2.36 ± 0.03 ^dD^	1.48 ± 0.02 ^dF^
	Sk	3.79 ± 0.01 ^eA^	0.29 ± 0.05 ^dD^	0.22 ± 0.01 ^eF^	0.32 ± 0.01 ^eE^	1.34 ± 0.01 ^eC^	3.58 ± 0.03 ^bB^	1.31 ± 0.04 ^eC^
	Oe	6.77 ± 0.09 ^bCD^	6.88 ± 0.08 ^aBC^	7.09 ± 0.09 ^aB^	6.41 ± 0.01 ^bE^	6.59 ± 0.01 ^aDE^	8.16 ± 0.03 ^aA^	5.62 ± 0.02 ^aF^
	Ie	7.23 ± 0.01 ^aB^	6.89 ± 0.11 ^aC^	6.79 ± 0.01 ^bC^	7.15 ± 0.01 ^aB^	6.07 ± 0.07 ^bD^	7.88 ± 0.02 ^aA^	5.49 ± 0.01 ^bE^
Chlorogenic Acid (mg/g)	Ep	0.59 ± 0.07 ^dE^	0.49 ± 0.01 ^dF^	0.79 ± 0.01 ^dC^	1.16 ± 0.01 ^dB^	0.73 ± 0.01 ^dD^	0.70 ± 0.00 ^dD^	1.28 ± 0.02 ^dA^
	Pc	0.10 ± 0.00 ^eD^	0.13 ± 0.01 ^dB^	0.14 ± 0.01 ^dA^	0.11 ± 0.01 ^eC^	0.10 ± 0.00 ^dDE^	0.09 ± 0.01 ^dE^	0.12 ± 0.00 ^eB^
	Sk	3.77 ± 0.01 ^cC^	3.94 ± 0.01 ^cB^	3.58 ± 0.01 ^cD^	3.61 ± 0.04 ^cD^	3.48 ± 0.01 ^cE^	3.58 ± 0.03 ^cD^	4.69 ± 0.02 ^cA^
	Oe	23.28 ± 0.20 ^aG^	29.68 ± 0.13 ^aE^	49.16 ± 0.07 ^bC^	27.95 ± 0.29 ^bF^	35.59 ± 0.09 ^aD^	54.73 ± 0.12 ^aA^	52.75 ± 0.17 ^aB^
	Ie	21.80 ± 0.28 ^bF^	24.05 ± 0.12 ^bE^	51.89 ± 0.10 ^aA^	28.40 ± 0.18 ^aC^	26.13 ± 0.25 ^bD^	41.14 ± 0.05 ^bB^	40.43 ± 0.10 ^bB^
Neochloroge-nic Acid (mg/g)	Ep	0.44 ± 0.01 ^dF^	0.45 ± 0.01 ^dE^	0.47 ± 0.01 ^dC^	0.51 ± 0.00 ^cB^	0.46 ± 0.01 ^dD^	0.50 ± 0.01 ^dB^	0.53 ± 0.01 ^dA^
	Pc	0.09 ± 0.01 ^eC^	0.11 ± 0.01 ^eA^	0.09 ± 0.00 ^eB^	0.09 ± 0.01 ^dC^	0.09 ± 0.01 ^eC^	0.09 ± 0.01 ^eC^	0.09 ± 0.01 ^eC^
	Sk	3.66 ± 0.01 ^cB^	3.63 ± 0.00 ^cC^	3.56 ± 0.01 ^cD^	3.51 ± 0.02 ^bE^	3.54 ± 0.00 ^cD^	3.54 ± 0.01 ^cD^	3.83 ± 0.10 ^cA^
	Oe	19.99 ± 0.05 ^aE^	21.41 ± 0.33 ^aD^	22.21 ± 0.10 ^aC^	19.62 ± 0.05 ^aF^	22.55 ± 0.03 ^aB^	22.21 ± 0.02 ^aC^	23.90 ± 0.07 ^aA^
	Ie	19.39 ± 0.01 ^bE^	19.01 ± 0.10 ^bF^	22.10 ± 0.00 ^bA^	19.67 ± 0.03 ^aD^	20.41 ± 0.10 ^bC^	20.99 ± 0.14 ^bB^	21.98 ± 0.16 ^bA^
Cryptochlorogenic acid (mg/g)	Ep	0.40 ± 0.01 ^dF^	0.40 ± 0.01 ^dF^	0.44 ± 0.10 ^dD^	0.48 ± 0.00 ^cB^	0.42 ± 0.00 ^dE^	0.47 ± 0.01 ^d C^	0.51 ± 0.01 ^dA^
	Pc	0.08 ± 0.01 ^eC^	0.10 ± 0.01 ^eA^	0.09 ± 0.01 ^eB^	0.08 ± 0.01 ^dC^	0.08 ± 0.00 ^eC^	0.08 ± 0.01 ^eC^	0.10 ± 0.00 ^eA^
	Sk	3.28 ± 0.00 ^cB^	3.26 ± 0.00 ^cC^	3.18 ± 0.01 ^cD^	3.10 ± 0.02 ^bE^	3.16 ± 0.00 ^cD^	3.17 ± 0.01 ^cD^	3.49 ± 0.01 ^cA^
	Oe	18.24 ± 0.10 ^aE^	34.19 ± 0.10 ^aA^	21.35 ± 0.14 ^aC^	18.09 ± 0.01 ^aE^	21.28 ± 0.09 ^aC^	20.75 ± 0.14 ^aD^	23.02 ± 0.11 ^aB^
	Ie	17.58 ± 0.13 ^bF^	17.36 ± 0.06 ^bG^	21.07 ± 0.18 ^bA^	18.00 ± 0.14 ^aD^	18.76 ± 0.01 ^bE^	19.63 ± 0.14 ^bC^	20.76 ± 0.12 ^bB^
3,5-di-O-caffeoylquinic acid (mg/g)	Ep	0.43 ± 0.01 ^dE^	0.43 ± 0.01 ^dE^	0.44 ± 0.01 ^dDE^	0.46 ± 0.00 ^dC^	0.44 ± 0.01 ^dD^	0.53 ± 0.01 ^dB^	0.55 ± 0.01 ^dA^
	Pc	0.09 ± 0.00 ^eA^	0.09 ± 0.00 ^eA^	0.09 ± 0.00 ^eA^	0.09 ± 0.01 ^eA^	0.09 ± 0.00 ^eA^	0.09 ± 0.01 d^eA^	0.07 ± 0.00 ^eB^
	Sk	3.51 ± 0.07 ^cB^	3.49 ± 0.00 ^cC^	3.48 ± 0.01 ^cD^	3.47 ± 0.01 ^cE^	3.48 ± 0.01 ^cD^	3.49 ± 0.01 ^cC^	3.55 ± 0.07 ^cA^
	Oe	17.70 ± 0.03 ^aC^	18.25 ± 0.02 ^aB^	18.30 ± 0.07 ^bB^	18.11 ± 0.04 ^bB^	19.14 ± 0.16 ^aA^	19.39 ± 0.07 ^aA^	18.37 ± 0.10 ^aB^
	Ie	17.61 ± 0.04 ^bC^	17.71 ± 0.03 ^bC^	18.43 ± 0.11 ^aA^	18.19 ± 0.04 ^aB^	18.28 ± 0.08 ^bB^	18.52 ± 0.09 ^bA^	18.25 ± 0.02 ^bB^

Note: Different lowercase letters within the same column denote significant differences among tissues, and different uppercase letters within the same row represent significant differences among varieties (*p* < 0.05). exocarp (Ep), parchment (Pc), silverskin (Sk), outer endosperm (Oe), inner endosperm (Ie). De’re 390 (DR390), De’re 394 (DR394), De’re 397 (DR397), De’re 401 (DR401), De’re 402 (DR402), Reyan No.3 (RY3), Reyan No.5 (RY5).

**Table 4:** Relative odor activity values of volatile compounds in green coffee beans (Oe+Ie) of coffee fruits from different varieties.

Number	Volatile Compounds	Sensory Threshold (µg/kg)	Relative Odor Activity Value (ROAV)													
			Oe							Ie						
			DR390	DR394	DR397	DR401	DR402	RY3	RY5	DR390	DR394	DR397	DR401	DR402	RY3	RY5
1	Methyl 2-phenylacetate	60	0.682	0.662	0.667	0.627	0.658	0.610	0.638	0.517	0.590	0.448	0.585	0.556	0.698	0.596
2	Methyl salicylate	40	0.666	0.628	0.530	0.538	0.471	1.033	1.068	0.587	0.761	0.496	0.552	0.528	1.029	1.164
3	2,3-Pentanedione	30	0.303	0.000	0.000	0.000	0.000	0.000	0.000	0.231	0.280	0.201	0.000	0.000	0.000	0.000
4	5-ethyldihydro-2(3H)-Furanone	200	0.588	0.473	0.000	0.000	0.000	0.000	0.000	0.449	0.388	0.315	0.000	0.000	0.000	0.000
5	Phenethyl alcohol	390	0.093	0.179	0.067	0.142	0.109	0.157	0.113	0.091	0.209	0.086	0.173	0.147	0.166	0.131
6	Nonanal	0.001	0.023	0.041	0.041	0.030	0.062	0.021	0.028	0.026	0.042	0.047	0.034	0.051	0.023	0.030
7	Benzaldehyde	350	0.130	0.198	0.137	0.279	0.092	0.130	0.118	0.108	0.210	0.150	0.286	0.081	0.125	0.126
8	(E)- 2-Nonenal	0.08	0.000	0.000	20.732	0.000	0.000	24.004	28.877	0.000	0.000	21.357	0.000	0.000	35.899	34.016
9	3-Methyl Butanoic acid	400	0.000	0.099	0.220	0.151	0.102	0.000	0.096	0.134	0.258	0.187	0.088	0.141	0.157	0.000
10	2,3,5-Trimethylpyrazine	1	100.00	100.00	100.00	100.00	100.00	100.00	100.00	100.00	100.00	100.00	100.00	100.00	100.00	100.00
11	2,5-Dimethylpyrazine	20	0.714	0.784	0.768	0.899	1.304	1.250	1.060	0.858	0.870	0.766	0.785	0.872	1.090	1.000
12	Pyrazine	177,000	0.001	0.001	0.001	0.001	0.001	0.001	0.001	0.001	0.001	0.001	0.001	0.001	0.001	0.001
13	Furfural	3000	0.010	0.009	0.007	0.003	0.005	0.004	0.007	0.008	0.010	0.004	0.004	0.006	0.004	0.008

Note: outer endosperm (Oe), and inner endosperm (Ie). Varieties De’re 390(DR390), De’re 394(DR394), De’re 397(DR397), De’re 401(DR401), De’re 402(DR402), Reyan No.3(RY3), Reyan No.5(RY5).

**Table 5:** Relative odor activity values of volatile compounds in coffee byproducts (Ep, Pc, and Sk) of coffee fruits from different varieties.

Number	Volatile Compounds	Sensory Threshold (µg/kg)	Relative Odor Activity Value (ROAV)																				
			Ep							Pc							Sk						
			DR390	DR394	DR397	DR401	DR402	RY3	RY5	DR390	DR394	DR397	DR401	DR402	RY3	RY5	DR390	DR394	DR397	DR401	DR402	RY3	RY5
1	Methyl hexoate	87	1.051	0.954	0.852	1.102	1.134	0.951	0.914	—	—	—	—	—	—	—	0.996	0.655	0.688	4.045	3.075	1.412	0.233
2	Methyl octylate	200	0.287	0.242	0.256	0.279	0.271	0.258	0.228	0.523	0.588	0.524	0.715	1.543	0.620	0.475	0.756	0.193	0.245	1.695	—	0.943	—
3	Methyl Caprate	4.3	—	—	—	4.430	5.000	3.623	3.113	—	—	—	—	—	—	—	14.903	7.600	9.839	50.438	72.910	18.768	30.342
4	Methyl 2-phenylacetate	60	1.626	1.193	1.091	1.349	1.411	1.454	1.407	—	—	—	—	—	—	—	0.358	—	—	—	—	8.684	6.927
5	Methyl salicylate	40	2.615	2.546	2.724	2.262	2.389	2.351	2.404	3.008	3.495	3.699	8.318	7.716	5.025	4.875	2.949	2.623	2.826	19.545	3.514	2.434	1.895
6	3-Hexanone	61	—	—	—	—	—	—	—	0.740	0.744	0.426	0.414	0.688	—	—	0.078	0.330	0.074	1.937	1.241	0.259	0.192
7	2-Heptanone	650	—	—	—	—	—	—	—	0.037	0.037	0.035	0.047	0.032	—	—	—	—	—	—	—	—	—
10	2-Heptanol	70	0.736	0.785	0.745	0.676	0.634	—	—	—	—	—	—	—	—	—	—	—	—	—	—	—	—
11	Linalool	0.17	—	0.000	64.491	85.901	88.336	98.317	96.432	—	—	—	—	—	—	—	—	—	—	—	—	—	—
12	Benzyl alcohol	5500	0.008	0.008	0.007	0.009	0.009	0.009	0.008	0.018	0.023	0.021	0.036	0.022	0.049	0.027	0.004	0.006	0.005	—	—	—	—
14	Phenethyl alcohol	390	0.132	0.275	0.262	0.247	0.311	0.249	0.233	0.389	0.318	0.077	0.508	0.195	0.700	0.721	0.207	0.290	0.204	1.359	2.044	0.825	0.673
15	Prenol	300	—	—	—	—	—	—	—	—	—	—	—	—	—	—	0.019	0.071	0.016	0.247	0.342	—	—
17	Hexanal	5	27.975	25.926	32.757	26.222	24.437	25.212	21.858	—	—	—	—	—	—	—	—	—	—	—	—	—	—
18	Nonanal	1000	0.004	0.005	0.011	0.008	0.010	0.009	0.008	0.283	0.158	0.089	0.126	0.114	0.067	0.187	0.022	—	—	—	0.073	0.028	0.029
19	Heptenal	550	0.240	0.226	0.228	0.220	0.241	0.210	0.250	—	—	—	—	—	—	—	—	—	—	—	—	—	—
20	Benzaldehyde	350	0.166	0.188	0.163	0.168	0.175	0.175	0.150	0.327	0.444	0.374	0.454	0.289	0.826	0.703	0.248	0.084	0.064	—	—	0.657	0.348
21	Octanal	0.7	182.734	165.344	169.910	—	—	192.230	154.957	—	—	—	—	—	—	—	—	—	—	—	—	—	—
22	Phenylacetaldehyde	4	7.822	6.944	8.056	9.762	10.751	6.586	4.918	—	—	—	—	—	—	—	—	—	—	—	—	—	—
23	(E)- 2-Nonenal	0.08	—	—	—	—	—	—	—	79.887	54.398	98.577	79.439	33.046	—	—	—	—	—	—	—	—	—
25	Octanoic acid	910	0.029	0.028	0.027	—	—	0.028	0.025	—	—	—	—	—	—	—	—	—	—	—	—	—	—
26	Acetic acid	34,000	0.002	0.002	0.002	0.002	0.002	0.002	0.002	—	—	—	—	—	0.002	0.004	—	—	—	—	—	0.003	0.003
27	3-Methyl Butanoic acid	400	—	—	—	—	—	—	—	—	—	—	—	—	0.240	0.220	—	—	—	—	—	0.401	0.373
28	2-Acetyl pyrrole	10,000	0.011	0.010	0.010	—	—	—	—	—	—	—	—	—	—	—	—	—	—	—	—	—	—
29	2,4-Di-tert-butylphenol	500	—	—	—	—	—	—	—	1.063	1.107	1.389	2.768	1.376	2.478	2.956	0.474	0.109	0.176	1.005	1.584	0.684	0.644
30	2,3-Dimethylpyrazine	2.5	32.025	26.049	30.299	29.841	31.263	29.122	28.415	—	—	—	—	—	—	—	—	—	—	—	—	—	—
31	2,3,5-Trimethylpyrazine	1	100.00	100.00	100.00	100.00	100.00	100.00	100.00	100.00	100.00	100.00	100.00	100.00	100.00	100.00	100.00	100.00	100.00	100.00	100.00	100.00	100.00
33	2,5-Dimethylpyrazine	20	0.491	0.478	0.698	0.683	0.802	0.411	0.423	1.391	3.148	2.927	4.626	0.661	4.850	3.950	0.370	0.577	0.491	3.636	7.162	2.544	2.266
34	5-Methyl-2-furaldehyde	6	18.609	19.753	19.989	19.471	19.966	18.366	16.758	—	—	—	—	—	—	—	—	—	—	—	—	—	—
35	2-pentyl- Furan	5.9	—	—	—	—	—	—	—	—	—	—	—	—	—	—	4.834	—	3.803	—	8.704	20.666	—

Note: Exocarp (Ep), parchment (Pc), silverskin (Sk). Varieties De’re 390(DR390), De’re 394(DR394), De’re 397(DR397), De’re 401(DR401), De’re 402(DR402), Reyan No.3(RY3), Reyan No.5(RY5).

## Data Availability

The original contributions presented in this study are included in the article/Supplementary Material. Further inquiries can be directed to the corresponding author.

## Supplementary Materials

The following supporting information can be downloaded at: https://www.mdpi.com/article/10.3390/foods15020269/s1, Table S1: Electronic nose chemical sensors and their corresponding sensitive substance types; Table S2: Volatile Components in Different Tissues (exocarp, parchment, silverskin, outer endosperm, and inner endosperm) of Coffee Fruit. Figure S1: Fresh weight (a) and moisture content (b) of different structural tissues in coffee fruit of different varieties. Figure S2: Flavor wheel analysis among the distinct tissues of coffee fruit of different varieties. References [100,101,102,103,104,105,106,107,108,109,110,111,112,113,106,114,115,116,117,118,119,120,121,122,102,123,124,125,126,127,128,129,130,131,132,120,133,134,135,136] are cited in the Supplementary Materials.
