# Supplementary material for: HPLC/GC–MS and Electronic Sensing Reveal Tissue-Wide Differences in Bioactive and Flavor Compound Distribution in Coffee Fruits Across Multiple Varieties
Source: Foods. 2026 Jan 12;15(2):269. doi: 10.3390/foods15020269 (PMC12840179; doi:10.3390/foods15020269)
Supplement: Supplementary file 1 [file foods-15-00269-s001.zip › foods-4027078-supplementary.pdf]

Supplementary Materials

Table S1- Electronic nose chemical sensors and their corresponding sensitivity types

Number	Sensor name	Performance description
MOS 1	W1C	Sensitive to aromatic components (benzene)
MOS 2	W5S	Sensitive to nitrogen oxides
MOS 3	W3C	Sensitive to aromatic components (amines)
MOS 4	W6S	Selective for hydrogen (hydride)
MOS 5	W5C	Sensitive to alkanes and aromatic components (short-chain alkanes)
MOS 6	W1S	Sensitive to methane (methyl compounds)
MOS 7	W1W	Sensitive to terpenoids and inorganic sulfides
MOS 8	W2S	Sensitive to alcohols, aldehydes and ketones
MOS 9	W2W	Sensitive to aromatic components and organic sulfides
MOS 10	W3S	Sensitive to alkanes (long-chain alkanes)

Table S2-1- Volatile compounds in the exocarp (Ep) of coffee fruits from different varieties

Types	Retention time	Compounds	Experimental retention indice	Literature retention indice	Relative content（μg/g）							Odor description
					DR390	DR394	DR397	DR401	DR402	RY3	RY5	
Esters 1	17.90	Methyl hexoate	1192	1197 [100]	0.298	0.269	0.223	0.302	0.289	0.292	0.291	Pineapple, Apricot
2	26.42	Methyl octylate	1394	1399 [100]	0.187	0.157	0.154	0.176	0.159	0.182	0.167	Oranges,Vegetables, Herbs
3	34.80	Methyl Caprate	1599	1604 [101]	—	—	—	0.060	0.063	0.055	0.049	Raw fruit
4	40.52	Benzyl acetate	1751	1743 [102]	0.052	0.049	0.040	—	—	—	—	Fresh, Cooked vegetables, Fruity aroma, Jasmine
5	41.39	Methyl 2-phenylacetate	1775	1779 [100]	0.318	0.232	0.197	0.255	0.248	0.308	0.309	Honey aroma, Patchouli
6	42.22	Methyl salicylate	1798	1798 [103]	0.341	0.330	0.328	0.285	0.280	0.332	0.352	Almond, Caramel, Minty, Spicy
7	42.69	Ethyl salicylate	1817	1834 [104]	—	—	0.079	0.088	0.067	0.121	0.131	Wintergreen, Minty, Floral, Fruity, Spicy
8	49.57	Methyl tetradecanoate	2017	2020 [104]	0.347	0.341	0.329	0.346	0.316	—	—	Winey, Honey
9	56.01	Methyl palmitate	2224	2218 [105]	—	—	—	—	—	0.173	0.198	Waxy
Ketones 1	13.22	Methylpropylketene	1067	N/A	0.041	0.035	0.031	—	—	—	—	/
2	36.00	Methylheptenone	1335	1346[106]	0.063	0.072	0.061	0.052	0.051	0.062	0.060	Citrus, Peppery, Strawberry
3	43.99	Geranylacetone	1849	N/A	0.022	—	—	0.040	0.042	0.038	0.041	Fruity
4	57.90	5-Hydroxy-4-octanone	2296	N/A	0.492	0.448	0.432	0.410	0.408	0.455	0.428	Sweet Cream, Cardamom
Alcohols 1	17.82	2-Heptanol	1307	1320 [107]	0.168	0.178	0.157	0.149	0.130	—	—	Citrus
2	30.32	Oct-1-en-3-ol	1473	1452 [106]	0.365	0.373	0.340	0.374	0.379	0.348	0.311	Herbal, Lavender

3	36.23	Linalool	1548	1556 [106]	—	—	0.033	0.046	0.044	0.059	0.060	Floral, Lavender, Lemon, Rose
4	41.34	3-Furancarbinol	1638	1649 [108]	0.071	0.063	0.051	—	—	0.069	0.090	Caramel
5	42.35	(E)-2-decen-1-ol	1813	1819 [109]	0.073	0.062	0.061	0.065	0.058	0.072	0.065	Fruity
6	45.40	Benzyl alcohol	1890	1898 [110]	0.151	0.141	0.124	0.164	0.151	0.169	0.155	Cooked Cherry, Roasted, rose
7	46.62	1,4-Butanediol	1926	N/A	0.285	0.315	0.270	—	—	—	—	/
8	46.64	Phenylethyl alcohol	1928	1925 [102]	0.168	0.348	0.307	0.304	0.355	0.343	0.333	Fruity, Honey, Clove, Rose, Winey
9	47.63	Nerol	1947	1932 [102]	0.029	0.034	0.034	—	—	0.030	0.030	Rose, Lemon
Aldehydes 1	10.32	Hexanal	918	932 [111]	0.456	0.420	0.493	0.413	0.358	0.445	0.400	Fresh, Green, Fatty
2	26.71	Nonanal	1401	1396 [106]	—	—	0.032	0.026	0.029	0.031	0.031	Fatty, Floral, Lemon
3	28.77	Heptenal	1346	1335 [112]	0.430	0.402	0.377	0.382	0.388	0.407	0.504	Almond, Fatty, Fruity, Spicy
4	32.53	Benzaldehyde	1543	1538 [106]	0.189	0.213	0.172	0.185	0.179	0.216	0.192	Almond, Caramel, Fruity
5	32.76	Octanal	1302	1289 [113]	0.417	0.375	0.358	—	—	0.475	0.397	Fruity
6	34.02	Decanal	1536	1523 [106]	—	—	—	0.048	0.051	—	—	Floral, Orange Blossom, Citrus
7	34.77	Phenylacetaldehyde	1617	1635 [114]	0.102	0.090	0.097	0.123	0.126	0.093	0.072	Sweet, Floral
Acids 1	44.32	Isovaleric acid	1699	N/A	0.073	0.062	0.061	0.067	0.062	0.064	0.054	Sweet, Acid, Berry
2	16.39	Octanoic acid	1172	1191 [115]	0.087	0.082	0.073	—	—	0.090	0.083	Acid, Cheesy, Fruity
3	29.08	Acetic acid	1446	1427 [111]	0.213	0.179	0.196	0.213	0.230	0.207	0.187	Spicy, vinegar
4	54.55	Nonanoic acid	2172	2152 [102]	0.042	0.036	0.040	0.039	0.036	0.041	0.039	Fatty, Acid
5	56.71	Myristic acid	2653	2670 [116]	—	—	—	0.041	0.043	0.040	0.042	Burnt, Cheesy
Hydrocarbons 1	10.83	Hexadiene	1012	N/A	0.073	0.060	0.057	0.060	0.053	—	—	/
2	25.31	2-Octene	1368	N/A	0.046	0.040	0.031	—	—	0.043	0.053	/
3	28.87	trans-2-nonene	1453	N/A	—	—	0.054	0.041	0.039	0.046	0.037	/

Furans 1	13.23	2,3-dihydro- Furan	1065	N/A	0.025	0.026	0.030	0.029	0.028	0.027	0.036	Pungent
2	33.12	5-Methyl-2-furaldehyde	1578	1588 [117]	0.364	0.384	0.361	0.368	0.351	0.389	0.368	Spicy, Sweet, Caramel
Pyrrole 1	46.81	2-Acetyl pyrrole	1942	1960 [118]	0.357	0.325	0.303	—	—	—	—	Nutty, Walnut, Bready
Pyrazines 1	29.50	2,3-Dimethylpyrazine	1336	1353 [118]	0.261	0.211	0.228	0.235	0.229	0.257	0.260	Nutty, Creamy, Cocoa, Caramel
2	30.03	2,3,5-Trimethylpyrazine	1422	1411 [110]	0.362	0.324	0.301	0.315	0.293	0.353	0.366	Cocoa, Potato, Roast
3	28.07	2,5-Dimethylpyrazine	1346	1328 [117]	0.032	0.031	0.042	0.043	0.047	0.029	0.031	Nutty, Roast

Note: "—" indicates below the detection limit, and "/" indicates that no relevant flavor description was found. "N/A" indicates that a reference retention index for this compound on a similar stationary phase (HP-innowax) is not available in the literature. De're 390(DR390), De're 394(DR394), De're 397(DR397), De're 401(DR401), De're 402(DR402), Reyan No.3(RY3),Reyan No.5(RY5).

Table S2-2- Volatile Compounds in the parchment (Pc) of coffee fruits from different varieties

Types	Retention time	Compounds	Experimental retention indice	Literature retention indice	Relative content（μg/g）							Odor description
					DR390	DR394	DR397	DR401	DR402	RY3	RY5	
Esters 1	16.42	Heptyl isobutyrate	1153	N/A	0.041	0.039	0.024	—	—	—	—	Fruity
2	24.67	3-Octyl 2-methylbutyrate	1353	N/A	0.057	0.071	0.072	0.045	0.090	—	—	Fruity
3	26.42	Methyl octylate	1394	1399 [100]	0.139	0.127	0.129	0.153	0.537	0.124	0.095	Fruity, Wine
4	30.68	Methyl nonanoate	1496	1500 [100]	0.220	0.217	0.230	0.146	0.157	0.269	0.602	Wine, Coconu
5	42.22	Methyl salicylate	1798	1798 [103]	0.160	0.151	0.182	0.356	0.537	0.201	0.195	Wintergreen, Cherry, Apple
6	42.52	Methyl laurate	1807	1815 [104]	—	—	—	—	0.693	0.138	0.208	Green Fruit Notes
7	49.56	Methyl tetradecanoate	2016	2020 [104]	0.294	0.202	0.132	0.189	0.141	0.271	0.479	Wine, Honey
8	56.01	Methyl palmitate	2224	2218 [106]	0.106	0.125	0.155	0.464	0.628	0.675	0.523	Waxy
9	62.55	Methyl stearate	2442	2422 [119]	0.141	—	—	—	0.223	—	0.132	Waxy
Ketones 1	19.65	3-Hexanone	1092	1072 [120]	0.060	0.049	0.032	0.027	0.073	—	—	Sweet fruit, Waxy
2	20.31	2-Heptanone	1170	1189 [110]	0.032	0.026	0.028	0.033	0.036	—	—	Cheesy, Banana, Coconut
3	37.94	Isophorone	1620	1607 [102]	0.042	0.034	0.034	0.041	0.049	—	—	Minty

Aldehydes 1	26.71	Nonanal	1401	1396 [106]	0.376	0.171	0.109	0.135	0.198	0.067	0.187	Fat, Floral, Green, Lemon
2	32.54	Benzaldehyde	1543	1538 [106]	0.152	0.168	0.161	0.170	0.176	0.289	0.246	Bitter Almond, Burnt Sugar, Cherry, Malt, Roasted
3	32.71	(E)- 2-Nonenal	1547	1548 [110]	0.085	0.047	0.097	0.068	0.046	—	—	Paper
4	37.21	Benzeneacetaldehyde	1662	1662 [112]	—	—	0.148	—	0.058	0.182	—	Floral，Fruity
5	43.61	2,4-dimethyl-Benzaldehyde	1738	1720 [121]	0.664	0.725	0.815	1.184	0.936	0.945	1.498	Almond
6	50.29	Pentadecanal	2038	2041 [122]	0.080	0.053	0.154	0.215	0.587	—	—	Waxy
Acids 1	26.49	Isobutyric acid	1584	1574 [111]	0.044	0.028	0.025	—	—	—	—	Creamy, Apple, Caramel,Bready
2	29.03	3-Methylbutanoic acid	1655	1670 [102]	—	—	—	—	—	0.096	0.088	Tobacco, Apple
3	29.05	Acetic acid	1446	1427 [111]	—	—	—	—	—	0.057	0.119	Vinegar
Alcohos 1	45.39	Benzyl alcohol	1890	1898 [110]	0.132	0.136	0.141	0.213	0.209	0.272	0.148	Cooked Cherry, Roasted, rose
2	46.63	Phenylethyl alcohol	1927	1925 [102]	0.202	0.134	0.037	0.212	0.132	0.273	0.281	Floral
Phenols 1	58.71	2,4-Di-tert-butylphenol	2315	2315 [117]	0.707	0.598	0.854	1.481	1.197	1.239	1.478	/
Pyzarines 1	26.50	Pyrazine	1231	1214 [117]	0.109	0.030	0.032	0.047	0.025	0.025	0.048	Corn with bitter note, Pungent, Sweet, Strong
2	30.03	2,3,5-Trimethylpyrazine	1422	1411 [110]	0.133	0.108	0.123	0.107	0.174	—	—	Cocoa, Potato, Roast
3	28.07	2,5-Dimethylpyrazine	1346	1328 [117]	0.037	0.068	0.072	0.099	0.023	0.097	0.079	Nutty, Roast
Hydrocarbons 1	19.95	3-methyl-1-Pentene	1241	N/A	0.042	0.361	0.047	0.397	0.058	—	—	/
2	31.06	4,5-Nonadiene	1506	N/A	0.056	0.096	0.051	0.060	0.023	0.059	0.060	Floral
Sulfide 1	30.37	2-Furylmethylsulfide	1489	1492 [123]	0.133	0.109	0.105	0.114	0.087	0.123	0.114	Caramel

Note: "—" indicates below the detection limit, and "/" indicates that no relevant flavor description was found. "N/A" indicates that a reference retention index for this compound on a similar stationary phase (HP-innowax) is not available in the literature. De’re 390(DR390), De’re 394(DR394), De’re 397(DR397), De’re 401(DR401), De’re 402(DR402), Reyan No.3(RY3),Reyan No.5(RY5).

Table S2-3- Volatile compounds in the silverskin (Sk) of coffee fruits from different varieties

Types	Retention time	Compounds	Experimental retention indice	Literature retention indice	Relative content（μg/g）							Odor description
					DR390	DR394	DR397	DR401	DR402	RY3	RY5	
Esters 1	17.90	Methyl hexoate	1192	1197 [103]	0.492	0.232	0.280	0.271	0.099	0.140	0.026	Fruity
2	24.66	3-Octyl 2-methylbutyrate	1353	N/A	0.134	—	—	0.034	0.019	0.027	0.025	/
3	26.42	Methyl octylate	1394	1399 [103]	0.859	0.157	0.229	0.261	—	0.215	—	Fruity, Wine
4	30.68	Methyl nonanoate	1496	1500 [103]	1.981	0.380	0.289	0.269	0.261	0.295	0.239	Wine, Coconut
5	32.93	Methyl 3-nonenoate	1552	N/A	0.179	0.069	—	0.060	—	—	—	Violet
6	34.80	Methyl Caprate	1599	1604 [101]	0.364	0.133	0.198	0.167	0.116	0.092	0.167	Raw fruit
7	34.90	Dimethyl succinate	1602	N/A	0.109	0.232	0.127	0.165	—	0.247	—	Winey, Fruity
8	41.39	Methyl 2-phenylacetate	1775	1779 [100]	0.122	—	—	—	—	0.594	0.532	Honey aroma, Patchouli
9	42.22	Methyl salicylate	1798	1798 [103]	0.67	0.427	0.529	0.602	0.052	0.111	0.097	Wintergreen, Cherry, Apple
10	42.52	Methyl laurate	1807	1815 [104]	0.722	0.350	0.594	0.344	0.355	0.424	0.379	Raw fruit
11	49.57	Methyl tetradecanoate	2016	2020 [104]	0.879	1.663	1.374	1.324	0.377	1.044	0.867	Wine, Honey
12	52.86	Methyl Pentadecanoate	2116	2099 [124]	0.781	0.559	0.778	0.594	0.382	1.118	0.504	Tobacco
13	56.01	Methyl palmitate	2224	2218 [105]	0.602	1.836	0.349	1.628	0.511	0.410	0.464	Waxy
14	62.55	Methyl stearate	2442	2422 [119]	0.408	0.543	0.978	0.319	0.168	0.286	0.298	Waxy
Ketones 1	16.63	3-Hexanone	1092	1079 [125]	0.027	0.082	0.021	0.091	0.028	0.018	0.015	Fruity, Waxy, Wine
2	37.88	Isophorone	1620	1607 [102]	0.034	0.013	0.028	—	—	—	—	Camphor, Minty
3	23.92	1-Penten-3-one	1326	1308 [126]	0.033	0.018	0.022	—	—	—	—	Waxy
4	16.92	2,3-Hexanedione	1156	1143 [127]	—	—	—	0.010	0.012	0.013	0.015	Creamy, Cheesy
5	45.66	4,6-Nonanedione	1897	N/A	—	0.024	—	—	—	0.020	0.029	/
6	53.29	Phytone	2134	2131 [128]	0.521	0.141	0.279	0.142	0.109	0.112	0.107	Tobacco
Aldehydes 1	26.71	Nonanal	1401	1396 [106]	0.124	—	—	—	0.027	0.032	0.037	Fat, Floral, Green, Lemon

2	32.53	Benzaldehyde	1543	1538 [106]	0.494	0.119	0.105	—	—	0.262	0.156	Bitter Almond, Burnt Sugar, Cherry, Malt, Roasted
3	43.62	2,4-dimethyl-Benzaldehyde	1728	1710 [121]	0.485	0.287	0.325	0.248	0.229	0.391	0.352	Almond
4	46.82	Tetradecanal	1932	1933 [122]	0.155	—	—	—	—	0.109	0.118	Cirtus, Camphor
5	50.30	Pentadecanal	2039	2041 [122]	0.571	1.099	0.46	0.797	0.372	0.411	0.263	Waxy
Acids 1	29.05	Acetic acid	1446	1427 [111]	—	—	—	—	—	0.126	0.130	vinegar
2	42.41	3-methyl-2-Butenoic acid	1800	1780 [129]	—	—	—	—	—	0.183	0.191	Tobacco
Alcohols 1	23.37	Prenol	1322	1328 [130]	0.032	0.087	0.023	0.057	0.038	—	—	Fruity
2	45.40	Benzyl alcohol	1890	1898 [110]	0.121	0.125	0.117	—	—	—	—	Boiled Cherries, Moss, Roasted Bread, Rose
3	46.64	Phenylethyl alcohol	1927	1925 [102]	0.458	0.461	0.372	0.408	0.295	0.367	0.336	Floral
Phenols 1	58.17	2,4-Di-tert-butylphenol	2315	2315 [117]	1.347	0.222	0.411	0.387	0.293	0.390	0.412	/
Pyrazines 1	30.03	2,3,5-Trimethylpyrazine	1422	1411 [110]	0.568	0.407	0.486	0.077	0.037	0.114	0.128	Cocoa, Earth, Must, Potato, Roast
2	28.07	2,5-Dimethylpyrazine	1346	1328 [117]	0.042	0.047	0.046	0.056	0.053	0.058	0.058	Nutty, Roast
Hydrocarbons 1	46.80	4,5-Nonadiene	1931	N/A	—	—	—	—	—	0.063	0.009	Floral
Furans 1	19.72	2-pentyl- Furan	1236	1231 [106]	0.162	—	0.105	—	0.019	0.139	—	Potato
2	24.66	2-tert-Butoxytetrahydrofuran	1353	N/A	—	—	—	—	—	0.015	0.013	Spicy
Sulfide 1	30.37	2-Furylmethylsulfide	1489	1492 [123]	0.163	0.117	0.139	0.039	0.039	0.033	0.030	Caramel

Note: "—" indicates below the detection limit, and "/" indicates that no relevant flavor description was found. "N/A" indicates that a reference retention index for this compound on a similar stationary phase (HP-innowax) is not available in the literature. De're 390(DR390), De're 394(DR394), De're 397(DR397), De're 401(DR401), De're 402(DR402), Reyan No.3(RY3),Reyan No.5(RY5).

Table S2-4- Volatile Compounds in the outer endosperm (Oe) of coffee fruits from different varieties

Types		Retention time	Compounds	Experimental retention indice	Literature retention indice	Relative content（μg/g）							Odor description
						DR390	DR394	DR397	DR401	DR402	RY3	RY5	
Esters	1	11.16	tert-butyl pentanoate	999	N/A	0.089	0.082	0.079	0.092	0.083	—	—	Ripe Fruit Aroma
	2	16.25	Pentyl valerate	1149	1150 [131]	—	—	—	0.092	0.078	0.065	0.052	Apple
	3	21.00	Tetrahydrofurfuryl propionate	1266	N/A	0.017	0.020	0.023	0.073	0.150	0.053	0.035	Fruity, Wine
	4	22.52	2-methylbutyl propanoate	1190	1173 [132]	0.019	0.022	0.021	—	—	—	—	Fruity, Wine
	5	24.64	3-Octyl 2-methylbutyrate	1352	N/A	—	—	—	0.060	0.045	0.067	0.078	Berry, Apple
	6	30.67	Methyl nonanoate	1496	1500 [100]	—	0.143	—	—	—	0.098	0.093	Wine, Coconut
	7	32.93	Methyl 3-nonenoate	1552	N/A	—	—	—	—	—	0.087	0.073	Melon Aroma
	8	41.40	Methyl 2-phenylacetate	1775	1779 [100]	0.126	0.147	0.164	0.138	0.109	0.101	0.121	Cocoa, Strawberry
	9	42.23	Methyl salicylate	1798	1798 [103]	0.082	0.093	0.087	0.079	0.052	0.114	0.135	Wintergreen, Cherry, Apple
	10	56.00	Methyl palmitate	2224	2218 [105]	1.077	1.785	1.189	1.125	0.852	0.825	0.874	Waxy
Ketones	1	16.41	5,6-Decanedione	1153	N/A	—	—	—	—	0.102	0.031	0.022	Watermelon, Melon, Rose
	2	16.92	2,3-Hexanedione	1156	1143 [127]	0.053	—	—	—	—	0.051	0.028	Reamy, Cheesy
	3	39.83	5-ethyldihydro -2(3H)-Furanone	1738	1726 [100]	0.362	0.350	—	—	—	—	—	Grassy, Sweet Aroma
	4	37.96	2(5H)-Furanone	1692	1712 [108]	—	—	—	0.016	0.023	0.028	0.021	Tobacco
	5	23.09	4-Hexen-3-one	1316	1296 [120]	0.019	0.015	0.035	0.046	—	—	—	Cirtus
	6	24.66	2,5-Heptanedione	1353	N/A	—	—	—	—	0.043	0.064	0.058	/
	7	24.09	2,3-Pentanedione	1093	1076 [117]	0.028	—	—	—	—	—	—	Buttery, Caramel, Cheesy
	8	25.29	2,5-dimethyl-3-Hexanone	1165	N/A	0.024	0.146	0.037	0.034	0.045	0.044	0.048	/
	9	25.70	2,2-Dimethyl-3-Heptanone	1377	N/A	0.031	0.045	—	—	0.081	0.076	0.079	Fruity

10	26.40	3-Pentanone	1056	1036 [125]	0.032	0.062	0.028	—	—	—	—	Spicy
11	37.53	Acetophenone	1671	1657 [124]	—	—	—	—	0.049	0.062	0.061	Hawthorn
Aldehydes 1	26.70	Nonanal	1401	1396 [106]	0.072	0.150	0.167	0.110	0.172	0.057	0.088	Fat, Floral, Green, Lemon
2	32.53	Benzaldehyde	1543	1538 [106]	0.140	0.256	0.197	0.358	0.089	0.126	0.130	Bitter Almond, Burnt Sugar, Cherry, Malt, Roasted
3	32.73	(E)- 2-Nonenal	1548	1548 [110]	—	—	0.068	—	—	0.053	0.073	Paper
4	37.26	Benzeneacetaldehyde	1664	1662 [112]	0.165	—	—	0.362	—	0.147	0.116	Floral, Fruity
5	43.62	2,4-dimethyl-Benzaldehyde	1781	1762 [133]	0.531	0.417	0.409	0.605	0.449	0.554	0.762	Almond
Alcohols 1	32.92	Glycol monoacetate	1552	N/A	—	—	—	0.025	0.012	0.027	0.019	Fruity
2	46.62	Phenylethyl alcohol	1926	1925 [102]	0.112	0.258	0.107	0.203	0.117	0.169	0.139	Floral
3	59.62	3-methyl-2-Butanol	1108	1091 [120]	0.022	0.021	0.025	—	—	—	—	Fruity
Acids 1	29.06	Acetic acid	1446	1427 [111]	—	—	—	0.043	0.038	—	—	Vinegar
2	37.61	3-methyl- Butanoic acid	1673	1680 [117]	—	0.147	0.361	0.221	0.113	—	0.121	Fruity
3	42.40	2,3-Dimethylacrylic acid	1822	1838 [134]	—	—	—	—	—	0.109	0.135	Tobacco
4	51.08	1,2-Benzenedicarboxylic acid	2063	N/A	1.24	1.421	1.263	—	—	—	—	Floral
Phenols 1	53.59	4-butyl- Phenol	2374	2360 [135]	0.023	0.019	0.020	—	—	—	—	/
2	58.71	3,5-Di-tert-butylphenol	2315	2328 [136]	0.178	0.167	0.183	—	—	—	—	/
Furans 1	28.13	Furfural	1435	1439 [111]	0.095	0.096	0.080	0.033	0.044	0.030	0.068	Almond
2	39.26	2-ethyl- Furan	1717	N/A	0.415	0.140	0.153	—	—	0.037	0.062	Minty, Tomato
Pyrazines 1	26.50	Pyrazine	1231	1214 [117]	0.330	0.354	0.460	0.448	0.319	0.285	0.338	Corn with bitter note, pungent, sweet, strong
2	30.03	2,3,5-Trimethylpyrazine	1422	1411 [110]	0.266	0.320	0.306	0.385	0.276	0.276	0.316	Cocoa, Earth, Must, Potato, Roast
3	28.07	2,5-Dimethylpyrazine	1346	1328 [117]	0.044	0.058	0.063	0.066	0.072	0.069	0.067	Nutty, Roast
Hydrocarbons 1	13.82	4-methyl-1-Undecene	1083	N/A	0.076	0.073	0.068	—	—	0.121	0.102	/
2	26.71	4,5-Nonadiene	1401	N/A	0.117	0.126	0.108	0.027	0.064	0.056	0.113	Floral

3	30.35	4,4-dimethyl-1-Pentene	1489	N/A	—	—	—	0.037	0.024	0.011	0.015	/
Sulfide 1	30.39	2-Furylmethylsulfide	1493	1492 [123]	0.147	0.158	0.169	0.160	0.119	0.098	0.119	Caramel

Note: "—" indicates below the detection limit, and "/" indicates that no relevant flavor description was found. "N/A" indicates that a reference retention index for this compound on a similar stationary phase (HP-innowax) is not available in the literature. De're 390(DR390), De're 394(DR394), De're 397(DR397), De're 401(DR401), De're 402(DR402), Reyan No.3(RY3),Reyan No.5(RY5).

Table S2-5- Volatile Compounds in the inner endosperm (Ie) of coffee fruits from different varieties

Types	Retention time	Compounds	Experimental retention indice	Literature retention indice	Relative content（μg/g）							Odor description
					DR390	DR394	DR397	DR401	DR402	RY3	RY5	
Esters 1	11.16	tert-butyl pentanoate	999	N/A	0.073	0.091	0.082	0.089	0.093	—	—	Ripe Fruit Aroma
2	16.25	Pentyl valerate	1149	1150 [131]	—	—	—	0.087	0.078	0.063	0.037	Apple
3	21.00	Tetrahydrofurfuryl propionate	1266	N/A	—	—	0.026	0.085	0.067	0.058	0.057	Fruity, Wine
4	22.52	2-methylbutyl propanoate	1190	1173 [132]	0.023	0.027	0.028	—	—	—	—	Fruity, Wine
5	24.64	3-Octyl 2-methylbutyrate	1352	N/A	—	—	—	0.056	0.067	0.039	0.037	Berry, Apple
6	30.67	Methyl nonanoate	1496	1500 [100]	0.078	0.121	0.098	—	—	0.087	0.102	Wine, Coconut
7	32.93	Methyl 3-nonenoate	1552	N/A	—	—	—	—	—	0.086	0.092	Melon Aroma
8	41.40		1775	1779 [100]	0.103	0.114	0.107	0.132	0.109	0.121	0.109	Cocoa, Strawberry
9	42.23	Methyl salicylate	1798	1798 [103]	0.078	0.098	0.079	0.083	0.069	0.119	0.142	Wintergreen, Cherry, Apple
10	56.00	Methyl palmitate	2224	2218 [105]	1.122	1.682	1.187	1.126	1.091	0.924	0.927	Waxy
Ketones 1	16.41	5,6-Decanedione	1153	N/A	—	—	—	—	0.106	0.056	0.052	Watermelon, Melon, Rose
2	16.92	2,3-Hexanedione	1156	1143 [127]	0.057	—	—	—	—	0.063	0.068	Reamy, Cheesy

3	39.83	5-ethyldihydro-2(3H)-Furanone	1738	1726 [100]	0.298	0.250	0.251	—	—	—	—	Grassy, Sweet Aroma
4	37.93	2(5H)-Furanone	1692	1712 [108]	—	—	—	0.032	0.037	0.026	0.029	Tobacca
5	23.09	4-Hexen-3-one	1316	1296 [120]	0.027	0.025	0.038	0.043	0.029	—	—	Cirtus
6	24.66	2,5-Heptanedione	1353	N/A	—	—	—	0.048	0.052	0.063	0.062	/
7	22.09	2,3-Pentanedione	1093	1056 [117]	0.023	0.027	0.024	—	—	—	—	Buttery, Caramel, Cheesy
8	25.29	2,5-Dimethyl-3-Hexanone	1368	N/A	0.023	0.046	0.032	0.042	0.039	0.052	0.056	/
9	25.70	2,2-Dimethyl-3-Heptanone	1377	N/A	0.035	0.046	—	—	0.079	0.073	0.069	Fruity
10	22.40	3-Pentanone	1056	1036 [125]	0.029	0.036	0.035	—	—	—	—	Spicy
11	37.53	Acetophenone	1671	1657 [124]	—	—	—	0.046	0.053	0.052	0.056	Hawthorn
Aldehydes 1	26.70	Nonanal	1401	1396 [106]	0.087	0.135	0.187	0.128	0.167	0.067	0.093	Fat, Floral, Green, Lemon
2	32.53	Benzaldehyde	1543	1538 [106]	0.125	0.237	0.209	0.376	0.093	0.126	0.135	Bitter Almond, Burnt Sugar, Cherry, Malt, Roasted
3	32.73	(E)- 2-Nonenal	1548	1548 [110]	—	—	0.068	—	—	0.083	0.083	Paper
4	37.26	Benzeneacetaldehyde	1664	1662 [112]	0.178	—	—	0.356	—	0.189	0.186	Floral, Fruity
5	43.62	2,4-dimethyl- Benzaldehyde	1781	1762 [133]	0.543	0.473	0.502	0.598	0.478	0.562	0.738	Almond
Alcohols 1	32.92	Glycol monoacetate	1552	N/A	—	—	—	0.027	0.019	0.032	0.025	Fruity
2	46.62	Phenylethyl alcohol	1926	1925 [102]	0.118	0.263	0.134	0.253	0.187	0.187	0.156	Floral
3	59.62	3-methyl-2-Butanol	1108	1091 [120]	0.024	0.021	0.027	—	—	—	—	Fruity
Acids 1	29.06	Acetic acid	1446	1427 [111]	—	—	—	0.024	0.065	—	—	Vinegar
2	37.61	3-methyl- Butanoic acid	1673	1680 [117]	0.153	0.178	0.332	0.297	0.132	0.184	0.181	Fruity
3	42.40	2,3-Dimethylacrylic acid	1822	1838 [134]	—	—	—	—	—	0.146	0.136	Tobacco
4	51.08	1,2-Benzenedicarboxylic acid	2063	N/A	1.36	1.41	1.37	—	—	—	—	Floral
Phenols 1	53.59	4-butyl- Phenol	2374	2360 [135]	0.026	0.028	0.031	—	—	—	—	/
2	58.71	3,5-Di-tert-butylphenol	2315	2328 [136]	0.154	0.156	0.163	—	—	—	—	/
Furans 1	28.13	Furfural	1435	1439 [111]	0.078	0.096	0.052	0.043	0.063	0.032	0.074	Almond
2	39.26	2-ethyl- Furan	1717	N/A	0.342	0.276	0.275	—	—	0.065	0.063	Minty, Tomato
Pyrazines 1	26.50	Pyrazine	1231	1214 [117]	0.364	0.423	0.445	0.378	0.365	0.278	0.338	Corn with bitter note, pungent, sweet, strong

2	30.03	2,3,5-Trimethylpyrazine	1422	1411 [110]	0.332	0.322	0.398	0.376	0.327	0.289	0.305	Cocoa, Earth, Must, Potato, Roast
3	28.07	2,5-Dimethylpyrazine	1346	1328 [117]	0.057	0.056	0.061	0.059	0.057	0.063	0.061	Nutty, Roast
Hydrocarbons 1	13.82	4-methyl-1-Undecene	1083	N/A	0.076	0.073	0.068	—	—	0.121	0.102	/
2	26.71	4,5-Nonadiene	1401	N/A	0.164	0.162	0.146	0.128	0.073	0.075	0.086	Floral
3	30.35	4,4-dimethyl-1-Pentene	1489	N/A	—	—	—	0.032	0.043	0.051	0.055	/
Sulfide 1	30.39	2-Furylmethylsulfide	1493	1492 [123]	0.165	0.164	0.125	0.176	0.187	0.138	0.139	Caramel

Note: "—" indicates below the detection limit, and "/" indicates that no relevant flavor description was found. "N/A" indicates that a reference retention index for this compound on a similar stationary phase (HP-innowax) is not available in the literature. De're 390(DR390), De're 394(DR394), De're 397(DR397), De're 401(DR401), De're 402(DR402), Reyan No.3(RY3),Reyan No.5 (RY5).

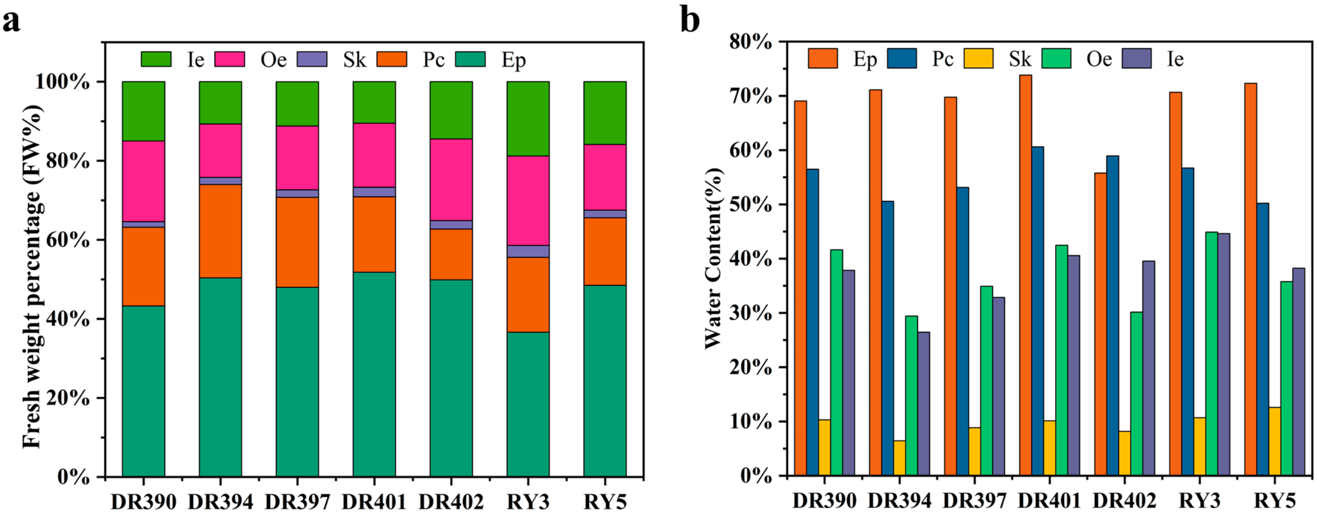

Figure S1 Fresh weight (a) and moisture content (b) of different structural tissues in coffee fruits from different varieties.

exocarp (Ep), parchment (Pc), silverskin (Sk), outer endosperm (Oe), and inner endosperm De’re 390(DR390), De’re 394(DR394), De’re 397(DR397), De’re 401(DR401), De’re 402(DR402), Reyan No.3(RY3),Reyan No.5 (RY5).

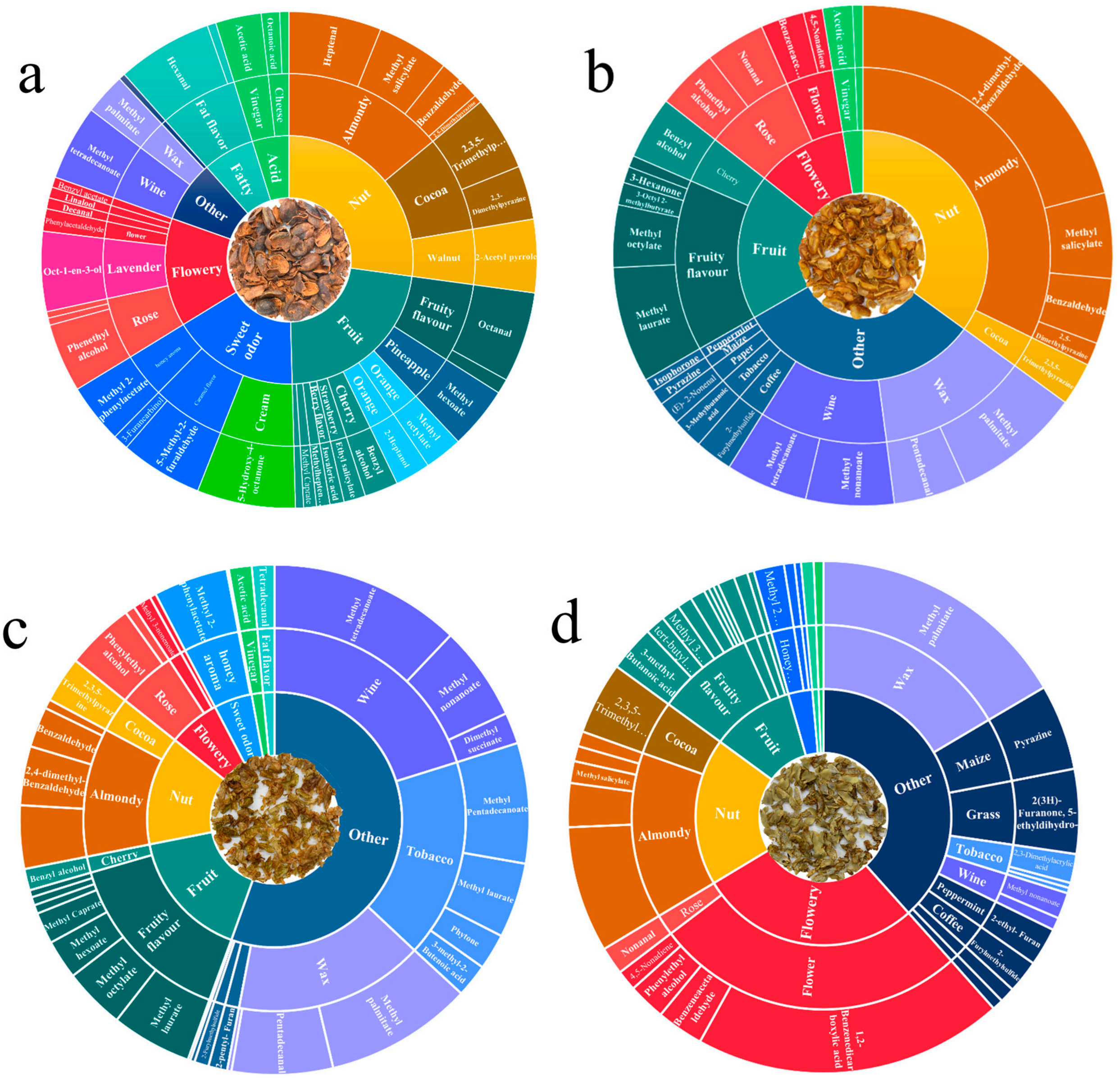

e

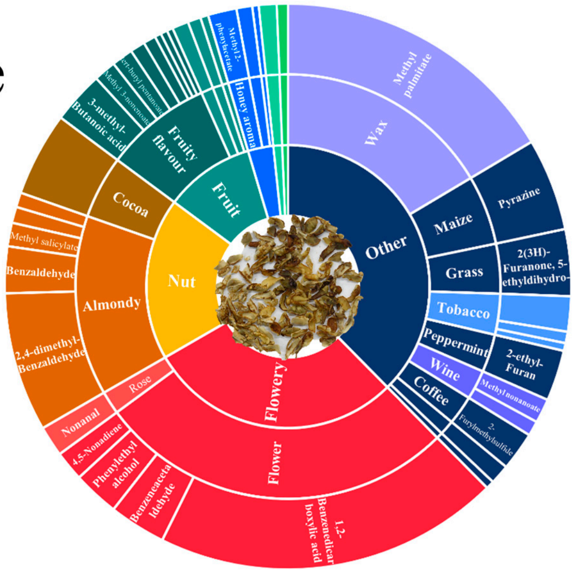

Figure S2 Flavor wheel analysis among the distinct tissues of coffee fruits from different varieties. a- exocarp flavor wheel, b- parchment flavor wheel, c- silverskin flavor wheel, d- outer endosperm flavor wheel, e- inner endosperm flavor wheel
